# An intravaginal ring for HIV pre-exposure prophylaxis: assessment of safety and pharmacokinetics in a randomized, placebo-controlled clinical trial

**DOI:** 10.3389/fcimb.2026.1955105

**Published:** 2026-10-06

**Authors:** Kathleen L. Vincent, John A. Moss, Mark A. Marzinke, Richard B. Pyles, Lauren Dawson, Trevelyn J. Olive, Irina Butkyavichene, Amalia E. Castonguay, John M. Cortez, Mariana Remedios-Chan, Patricia Galvan, Sofia Rivera, Simon Webster, Manjula Gunawardana, Amy P. Adler, Christina M. Ramirez, Hans M. L. Spiegel, Craig W. Hendrix, Peter A. Anton, Marc M. Baum

**Affiliations:** 1 Department of Obstetrics and Gynecology, University of Texas Medical Branch, Galveston, TX, United States; 2 Department of Chemistry, Oak Crest Institute of Science, Monrovia, CA, United States; 3 Division of Clinical Pharmacology, Department of Medicine, Johns Hopkins University, Baltimore, MD, United States; 4 Department of Pathology, Johns Hopkins University, Baltimore, MD, United States; 5 Department of Pediatrics, University of Texas Medical Branch, Galveston, TX, United States; 6 Department of Microbiology and Immunology, University of Texas Medical Branch, Galveston, TX, United States; 7 Jumpstart Research Consulting, LLC, Santa Fe, NM, United States; 8 Department of Biostatistics, UCLA Fielding School of Public Health, University of California, Los Angeles (UCLA), Los Angeles, CA, United States; 9 Kelly Government Solutions, Contractor to Division of AIDS, National Institute of Allergy and Infectious Diseases, National Institutes of Health, Rockville, MD, United States

**Keywords:** emtricitabine, HIV prevention, intravaginal ring, pharmacokinetics, PrEP, tenofovir disoproxil fumarate

## Abstract

**Introduction:**

In Eastern and Southern Africa, 63% of new HIV infections occurred in women and girls, with adolescent girls and young women representing a particularly vulnerable subpopulation. Effective vaginal products for HIV prevention are desirable because they are underrepresented in the currently approved portfolio and have attributes motivating product use. In this trial, we evaluated an innovative intravaginal ring (pod-IVR) as a platform for HIV pre-exposure prophylaxis (PrEP).

**Objective:**

To measure the safety and multicompartment pharmacokinetics (PKs) of a pod-IVR delivering tenofovir disoproxil fumarate (TDF) combined with emtricitabine (FTC) in healthy women over 28 days.

**Methods:**

The Phase 1 clinical trial consisted of a randomized order, controlled, double-blinded, crossover design. Ten women received either an active TDF-FTC pod-IVR or a placebo pod-IVR for 28 days, determined by 1:1 randomization, followed by product crossover for another 28 days. Safety was assessed in terms of adverse events related to product use, colposcopic examination, cervicovaginal epithelium thickness measurement by optical coherence tomography, and culture-independent assessment of the vaginal microbiome. Drug and/or drug metabolite concentrations were measured in cervicovaginal fluid (CVF), vaginal tissue, rectal tissue, rectal fluid, and plasma.

**Results:**

There was no evidence of a statistically significant difference in related adverse-event counts between study products. There also was no evidence suggesting the study product altered the vaginal microbiome systematically or reproducibly. Systemic plasma exposure to tenofovir (TFV) and FTC was low, consistent with topical delivery. High median CVF concentrations of TFV (38.9 ng mg^-1^; 135×10^3^ fmol mg^-1^) and FTC (239 ng mg^-1^; 966×10^3^ fmol mg^-1^) were measured. Median vaginal tissue concentrations of the anabolites responsible for inhibition of HIV reverse transcriptase exceeded our target concentrations: TFV diphosphate 0.651×10^3^ fmol mg^-1^ and FTC triphosphate 0.180×10^3^ fmol mg^-1^. Drug concentrations in rectal tissue samples generally were below the analytical lower limits of quantification, but were quantifiable in rectal fluids, 130 (TFV) and 300 (FTC) times lower than in CVF.

**Conclusions:**

The TDF-FTC pod-IVRs were safe and well-tolerated over the 28 days of consecutive use. The PK data met the target benchmarks associated with protection from vaginal HIV infection.

**Clinical trial registration:**

https://clinicaltrials.gov/study/NCT03255915, identifier NCT03255915.

## 1 Introduction

In 2025, an estimated 1.2 million people became newly infected with HIV, a 43% decline from 2010 (UNAIDS, 2026), largely attributable to successful implementation of biomedical prevention and treatment strategies. In Eastern and Southern Africa, 63% of new HIV infections occurred in women and girls (UNAIDS, 2025). Adolescent girls and young women (AGYW) aged 15–24 years represent a particularly vulnerable subpopulation, with an estimated average of 570 new infections per day in 2024 (UNAIDS, 2025). Accelerating the decline in global HIV infection rates will require HIV-1 pre-exposure prophylaxis (PrEP) approaches that target at-risk subpopulations. For AGYW, product diversity and innovation are widely regarded as key factors in motivating adoption (Shapley-Quinn et al., 2019; Schmidt et al., 2025). Vaginal products are desirable because they have the potential of being discreet, portable, and woman-controlled, while limiting systemic exposure to the drug(s) and their metabolites (Van Der Straten et al., 2017; Ridgeway et al., 2022; Friedland et al., 2025). They are, however, underrepresented in the currently approved portfolio of HIV prevention products. Intravaginal rings (IVRs) (Moss et al., 2014) are an appealing long-acting product class because they reduce the adherence burden associated with frequent dosing (Kruse et al., 1991; Quraishi and David, 2000; Sershen and West, 2002; Kutilek et al., 2003; Bhanji et al., 2004; Haycox, 2005; Small and Dubois, 2007; Yeaw et al., 2009), as required by the currently approved regimens of once daily oral Truvada^®^ (tenofovir disoproxil fumarate combined with emtricitabine, TDF-FTC) and Descovy^®^ (tenofovir alafenamide-FTC).

Two large Phase III clinical trials demonstrated that a monthly IVR delivering the non-nucleoside reverse transcriptase inhibitor dapivirine (DPV) was a safe and effective HIV-1 PrEP regimen (Baeten et al., 2016; Nel et al., 2016). The HOPE open-label extension study in South Africa and Uganda determined HIV-1 incidence among DPV IVR users to be 62% lower than the simulated placebo rate (Nel et al., 2021). Several clinical trials found that African AGYW were more likely to discontinue product use, but preferred the DPV IVR over oral HIV-1 PrEP (Nair et al., 2023; Fonner et al., 2025). First approved in 2021 by the Medicines Control Authority of Zimbabwe, the monthly DPV IVR has a total of 12 approvals, along with 2 pending in Ghana and Ethiopia as of January 2026 (Prepwatch, 2026), an important milestone in extending the HIV-1 PrEP product portfolio to long-acting, vaginal delivery options.

We have developed an innovative IVR technology platform enabling multiple agents to be delivered simultaneously. The so-called pod-IVR consists of up to 10 polymer-coated, single-drug solid tablets, “pods,“ embedded in an unmedicated ring scaffold, with delivery channels exposing a predetermined surface area of pods to cervicovaginal fluid (CVF) (Baum et al., 2012). Consequently, each pod independently delivers the drug at a target, controlled rate, often with zero order kinetics. The release characteristics on a per-pod basis can be tuned by varying the polymer coating and delivery channel properties. The pod-IVR design enables rapid development and *in vivo* evaluation of a wide range of formulations, including the controlled delivery of salts (Moss et al., 2012; Moss and Baum, 2014; Baum et al., 2015; Moss et al., 2016, 2026), biologics (Gunawardana et al., 2014a; Zhao et al., 2017), and live probiotic bacteria (Gunawardana et al., 2014b) that are challenging to achieve with other IVR technologies.

We have demonstrated that a pod-IVR delivering TDF and FTC, the combination used in oral Truvada^®^, completely protected normally cycling pig-tailed macaques against SHIV162p3 infection in a rigorous, repeat low-dose, vaginal challenge model (Srinivasan et al., 2016). We subsequently conducted a 7-day, crossover, open-label Phase 1 clinical trial evaluating the safety, pharmacokinetics (PKs), and acceptability of pod-IVRs delivering three ARV regimens, including TDF-FTC and a triple combination of TDF-FTC with maraviroc (Guthrie et al., 2018; Vincent et al., 2018a, 2018). The current report details the results from a follow-on Phase 1 clinical trial evaluating the TDF-FTC pod-IVR against placebo for 28 days using a crossover design.

## 2 Materials and methods

### 2.1 Ethics statement

All human research was approved by the University of Texas Medical Branch Institutional Review Board (IRB #17-0131), conducted according to the Declaration of Helsinki, and registered in clinicaltrials.gov (NCT03255915). All participants provided written informed consent prior to any study procedures.

### 2.2 Study product

Drug substances (TDF and FTC) labeled for human use were purchased from commercial vendors with a Drug Master File (DMF) registered with the US FDA. Active (TDF-FTC) and placebo IVRs with the characteristics shown in **Table 1** were manufactured as described in Vincent et al (Vincent et al., 2018b), and the references cited therein. Residual drug mass in IVRs after 28 d of use was measured by high-performance liquid chromatography (HPLC) according to published methods (Moss and Baum, 2014; Baum et al., 2015), and used to calculate *in vivo* TDF and FTC release rates.

**Table 1:** Intravaginal ring characteristics.

IVR type	Characteristic
TDF-FTC (study)	TDF pods: 4 per IVR; 180 mg TDF
	TDF pod composition: 99.5% w/w TDF; 0.5% w/w sodium stearyl fumarate
	TDF pod delivery channels: 3×1.5 mm diameter
	FTC pods: 3 per IVR; 135 mg FTC
	FTC pod composition: 99.5% w/w FTC; 0.5% w/w sodium stearyl fumarate
	FTC pod delivery channels: 1×0.5 mm diameter^1^
Placebo	placebo pods: 7 per IVR; 315 mg
	placebo pod composition: 99.5% w/w microcrystalline cellulose; 0.5% w/w sodium stearyl fumarate

^1^ Diameter indicated is punch cutting edge diameter used to create delivery channels in silicone IVR.

### 2.3 Clinical trial design

The Phase 1 clinical trial, PrEP-Pod-IVR-02 (DAIDS Protocol Number: 12032), consisted of a controlled, double-blinded, crossover design with participants randomized (1:1) to order of placebo or active to assess the safety and pharmacokinetics (PKs) of an intravaginal ring (IVR) delivering tenofovir disoproxil fumarate (TDF) in combination with emtricitabine (FTC) over 28 days. The trial was carried out under IND 135459 at the University of Texas Medical Branch, at Galveston over 2 years. The first participant was enrolled in October 2018, and the last participant visit occurred in September 2020. The trial design and study visits are summarized in **Figure 1**.

**Figure 1:**
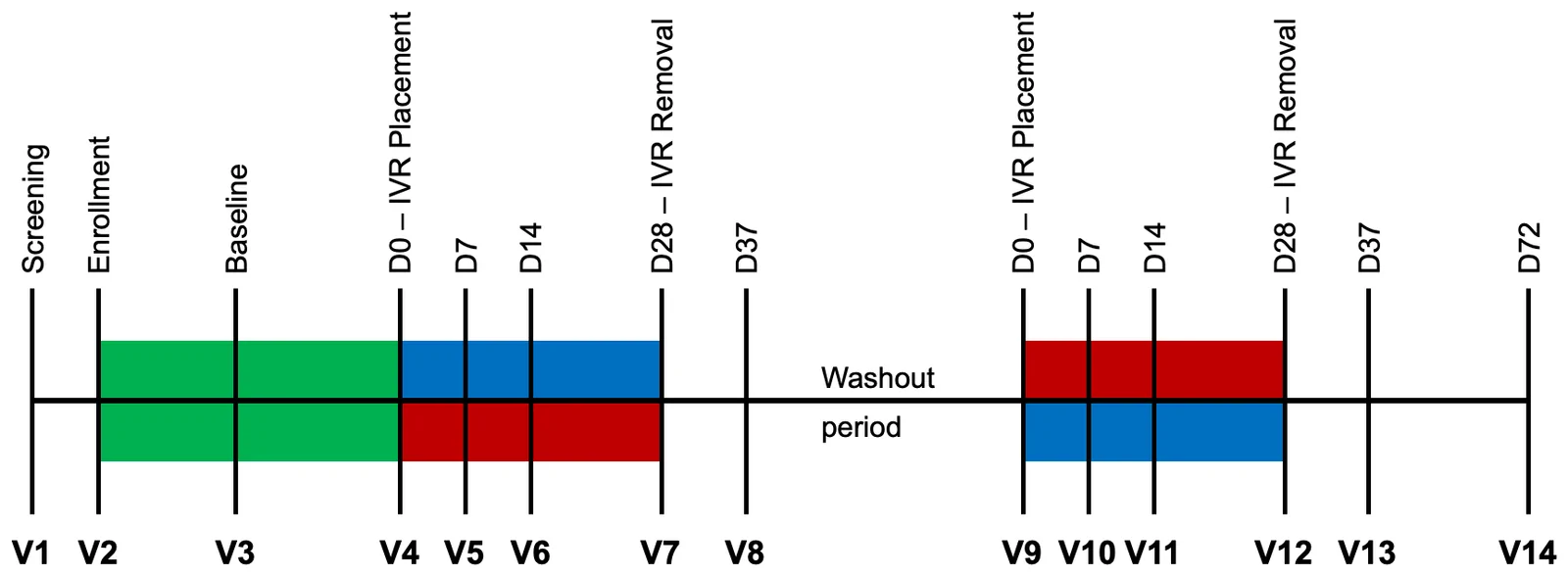
PrEP-Pod-IVR-02 Trial design and study visits. Stage 1 (green), V2-V4, consists of the baseline phase (no IVR); Stage 2 randomized 1:1 with placebo (blue, A group) and active (red, B group) IVRs, V4-V7, and Stage 3 crossover to active (red, A→B) and placebo (blue, B→A) IVRs, V9-V12.

Participants (*N* = 10) first completed a 28-day baseline assessment period without IVR use (**Figure 1**, green). They were then randomized 1:1 to one of two treatment sequences in a two-period crossover design: placebo IVR followed by TDF-FTC IVR (A→B) or TDF-FTC IVR followed by placebo IVR (B→A). Each IVR was used for 28 days, with a washout period of at least 2 weeks between treatment periods. Thus, all participants received both the active and placebo IVRs, with five participants assigned to each treatment sequence. Adverse events were recorded with daily diaries and during study visits.

Urine pregnancy testing was conducted at all study visits. Testing for sexually transmitted infections (*i.e.*, HIV-1, syphilis, GC/CT) and safety labs (CBC with differential and PLT, ALT, AST, BUN, creatinine) were performed at screening and repeated on the day of IVR insertion and removal; HIV testing also was performed 2 weeks after IVR removal and at the final study visit. Additional testing for hepatitis (*i.e.*, HBV and HCV) and a PT/INR was performed at screening. Urinalysis and wet mounts were performed at baseline and as needed. The clinical procedures and sample collections (V2-13, **Figure 1**, unless otherwise specified) were performed as described previously (Vincent et al., 2018b) and are included under the Supplementary Materials.

### 2.4 Trial endpoints

The endpoints of PrEP-Pod-IVR-02 were:

- Safety based on adverse events (AEs) Grade 2 or higher as defined by the Division of AIDS Table for Grading the Severity of Adult and Pediatric Adverse Events (DAIDS, 2017c), Addendum 1 Female Genital Grading Table for Use in Microbicide Studies (DAIDS, 2017a), and/or Addendum 3 Rectal Grading Table for Use in Microbicide Studies (DAIDS, 2017b); number of colposcopy findings; vaginal tissue histology; mucosal safety based on vaginal epithelial thickness measured by optical coherence tomography (OCT); cytology *via* microscopy (CVL and IVR wash); microbiology (*e.g.*, pH, Nugent score, culture-independent vaginal microbiome analysis),
- Pharmacokinetics, TDF and FTC drug, and/or drug metabolite, concentrations in cervicovaginal fluid (CVF), cervicovaginal lavage (CVL), vaginal tissue (VT), rectal tissue (RT), rectal fluid (RF), and plasma; adherence to IVR use based on CVF PK drug and drug metabolite concentrations; and residual drug in returned, used IVRs.

### 2.5 Enrollment

The study population consisted of healthy pre-menopausal females (born female) 18–45 years of age, in general good health and, if willing, their male sexual partner(s). Participants were HIV-1 seronegative at screening, at low risk of sexually transmitted infections (STIs), and were protected from pregnancy (systemic hormonal methods, IUDs, or sterilization) during the study. Participants agreed to abstain from use of vaginal products from enrollment to the final study visit and to abstain from receptive oral, vaginal, or anal intercourse during the first week after each IVR insertion and for 2 days before and 7 days after biopsy procedures.

Women who were pregnant, less than 3 months post-partum, lactating, or intended to become pregnant during the period of study participation were excluded. Participant-reported symptoms and/or clinical or laboratory diagnosis of active rectal or reproductive tract infection requiring treatment per current CDC guidelines or symptomatic urinary tract infection (UTI) led to exclusion from the trial. Other exclusion criteria included: significant laboratory abnormalities at screening, planned use of an IVR (other than the study product), known HIV-infected partners, history of autoimmune disease or toxic shock syndrome, and abnormalities of the cervical, vaginal, or colorectal mucosa.

### 2.6 Safety measures

#### 2.6.1 Clinical observations and analyses

Adverse events (AEs) were reviewed at all study visits, along with colposcopy, vaginal pH, and Nugent scores (Nugent et al., 1991) at V2 to V13. Histology was performed on vaginal tissue specimens collected on V7, V12, and V13 (**Figure 1**). Optical coherence tomography (OCT) was used to monitor vaginal epithelial thickness and microstructure according to published methods (Vincent et al., 2008, 2009). Briefly, a 3 mm OCT probe was inserted into the vagina to capture images of the cervix and fornix with the vaginal speculum in place. The speculum subsequently was removed for collection of vaginal images. As in previous studies (Vincent et al., 2008, 2009, 2013), the probe was placed in contact with the vagina, fornix, and cervix to collect three images each of the following sites: anterior (12 o’clock), posterior (6 o’clock), left (3 o’clock), and right (9 o’clock) vagina at the level of the mid-vagina; anterior (12 o’clock), posterior (6 o’clock), left (3 o’clock), and right (9 o’clock) vaginal fornices; and face of cervix at 12, 3, 6, and 9 o’clock. Typically, 3 replicate images from each of these 12 predetermined locations were obtained for each participant for a total of 36 images (3×4×3) per visit. The images were analyzed on a computer monitor at a later time for measurement of epithelial thickness using a validated scoring system (Vincent et al., 2008, 2009, 2013). Data were analyzed as means and standard deviations (*SD*s) of the 36 measurements per anatomic compartment.

#### 2.6.2 Culture-independent, quantitative analysis of vaginal microbiota

Vaginal microbiome (VMB) community structure was measured quantitatively for all participants at each study visit (V1-13) using a customized quantitative polymerase chain reaction (qPCR) array described previously (Rose et al., 2012; Pyles et al., 2014), including in a prior pod-IVR clinical trial involving delivery of TDF and FTC (Vincent et al., 2018b). The array uses a 96-well format and targets 46 distinct key vaginal microbiota to the species level along with several housekeeping genes. As in our previous clinical trial, aliquots of fluid from calcium alginate swabs mixed 1:1 with external lysis solution (Roche Diagnostics USA, Indianapolis, IN) were stored at -80 °C. Microbial DNA was extracted and purified using the MagNAPure 96 System (Roche Diagnostics USA, Indianapolis, IN), according to the manufacturer’s instructions.

### 2.7 Pharmacokinetic measures

Pharmacokinetic assay validation was performed in accordance with the FDA Guidance for Industry, Bioanalytical Method Validation recommendations and met all acceptability criteria (US FDA, 2001). Isotopically labeled internal standards for all compounds (except CVL samples, *vide infra*) were used in the determination of drug concentrations in all specimen sources.

#### 2.7.1 Analysis of cervicovaginal fluid, vaginal tissue, rectal fluid, rectal tissue, and plasma samples

Concentrations of tenofovir (TFV), TFV diphosphate (TFV-DP), and FTC were determined as in our prior clinical trial (Vincent et al., 2018b), using established liquid chromatographic-tandem mass spectrometric (LC-MS/MS) methods (Hendrix et al., 2013, 2016) at Johns Hopkins University. As with TFV-DP, FTC triphosphate (FTC-TP) was measured by indirect anabolite quantitation *via* isolation of FTC-TP and subsequent enzymatic conversion to FTC, prior to solid-phase extraction and drug quantitation by LC-MS/MS. The anabolites TFV-DP and FTC-TP were only analyzed in tissue homogenate samples, while TFV and FTC were measured in all matrices.

The lower limits of quantification for these methods were as follows: CVF and rectal fluid (RF), TFV (0.25 ng/sample), FTC (1.0 ng/sample); tissue homogenate, TFV (0.05 ng/sample), TFV-DP (5 fmol/sample), FTC (0.25 ng/sample), FTC-TP (50 fmol/sample); plasma, TFV (0.31 ng mL^-1^), FTC (0.31 ng mL^-1^). CVF, RF, and tissue samples were reported as ng mg^-1^ or fmol mg^-1^, following normalization to net biopsy or CVF weight.

#### 2.7.2 Measurement of cervicovaginal fluid dilution in lavage samples

The collection of CVL samples was performed by instilling a sterile phosphate-buffered saline solution (3 mL) containing LiCl (10 mM) *via* sterile catheter and lavaging 5 times. The added LiCl allowed dilution of the collected CVF to be calculated, and accounted for, using a method described elsewhere (Churchman et al., 2016; Vincent et al., 2018b) at Oak Crest Institute of Science.

#### 2.7.3 Analysis of cervicovaginal lavage samples

Separation of TDF, mono-isoproxil TFV intermediate (POC-TFV), and TFV in CVL samples was carried out on an Agilent XDB-C18 (2.1 × 50 mm; 3.5 µm particle size) column equipped with an Agilent C8 (2.1 × 12.5 mm; 5 µm particle size) guard column. The following gradient program was used (A: 0.1% v/v formic acid in water; B: 0.1% v/v formic acid in acetonitrile): 0.25 min 100% A; 1.25 min ramp from 100:0 A:B to 70:30 A:B; 1.0 min ramp from 70:30 A:B to 50:50 A:B; 1.0 min hold at 50:50 A:B; 1.5 min ramp from 50:50 A:B to 95:5 A:B; 0.5 min ramp from 95:5 A:B to 100:0 A:B (total run time 5.5 min). The analyte retention times were: TFV, 0.27 min; POC-TFV, 1.83 min; MVC, 2.38 min; and TDF, 2.60 min. The measured transition ions, m/z, in positive ESI mode were as follows: for TFV, 288.1 atomic mass units (amu) (parent) and 176.2 amu (product); for POC-TFV, 404.1 amu (parent) and 176.1 amu (product); for tenofovir disoproxil, 520.0 amu (parent) and 270.0 amu (product); for maraviroc (internal standard, IS, for TDF and POC-TFV), 514.7 amu (parent) and 280.6 amu (product); and for TFV-*d_6_* (IS), 293.1 amu (parent) and 181.2 amu (product).

Separation of FTC was carried out on a Waters Atlantis T3 (2.1 × 50 mm; 5 µm particle size) column equipped with a Waters Atlantis T3 (2.1 × 10 mm; 5 µm particle size) guard column. The following gradient program was used (A: 0.1% v/v formic acid in water; B: 0.1% v/v formic acid in acetonitrile): 0.25 min 100% A; 1.25 min ramp from 100:0 A:B to 70:30 A:B; 1.0 min ramp from 70:30 A:B to 50:50 A:B; 1.0 min hold at 50:50 A:B; 1.5 min ramp from 50:50 A:B to 95:5 A:B; 0.5 min ramp from 95:5 A:B to 100:0 A:B; 1.0 min hold at 100:0 A:B (total run time 6.5 min). The FTC retention time was 1.63 min. The measured transition ions, m/z, in positive ESI mode were as follows: for FTC, 248.0 atomic mass units (amu) (parent) and 130.0 amu (product); and for FTC-^13^C, ^15^N_2_ (IS), 251 amu (parent) and 133 amu (product).

The lower limits of quantification for these methods were as follows: TDF (5.0 ng/sample), POC-TFV (1.0 ng/sample), TFV (12 ng/sample), and FTC (10 ng/sample). The CVL samples were analyzed at Oak Crest Institute of Science.

### 2.8 Statistical analysis and data visualization

All data processing, visualization, and statistical analyses were performed using GraphPad Prism (version 11.0.0; GraphPad Software, Inc., La Jolla, CA) and RStudio 2025.09.1 + 401. Unless otherwise stated, concentration data are summarized as medians with interquartile ranges (IQRs), while concentration-time plots are presented as means ± standard deviations (*SD*s). Unless otherwise specified, all statistical tests were two-sided, with statistical significance defined as *P* < 0.05.

Values below the assay lower limit of quantification (LOQ) were treated as left-censored. For descriptive summaries in tables, analyte concentrations that were below the assay LOQ for samples collected while the IVR was in place were imputed using Equation 1:

(1) $$C_{LLQ}=\frac{\text{LOQ}}{2\times \tilde{m}}$$

where, *C_LLQ_* is the analyte LOQ in concentration units, LOQ is the assay limit of quantification on a per sample basis, and $\tilde{m}$ is the median sample mass (for swab-collected fluid and tissue biopsies) or the median sample volume (for lavage-derived samples after dilution correction), consistent with prior work (Vincent et al., 2018b). The proportion of quantifiable samples (% > LOQ) is reported for each analyte and matrix.

Adverse event counts were analyzed using a Poisson model appropriate for the randomized two-period crossover design. Participants were randomized to one of two treatment sequences: placebo IVR followed by active IVR (A→B) or active IVR followed by placebo IVR (B→A), where A denotes placebo and B denotes the active TDF-FTC IVR.

For participant *i* assigned to sequence A→B, let *X_ia_​* and *X_ib_*​ denote the numbers of adverse events observed during the placebo and active IVR periods, respectively. Conditional on the participant-specific effect, these counts were assumed to be independently Poisson-distributed, with expected values according to Equation 2:

(2) $$E\left(X_{ia}\right)=\exp\left(\beta 1i+\lambda +\delta \right),\;E\left(X_{ib}\right)=\exp\left(\beta 1i-\lambda -\delta \right),\;i=1,\ldots \text{na}$$

For participant *j* assigned to sequence B→A, let *Y_ja_* and *Y_jb_*​ denote the numbers of adverse events observed during the placebo and active IVR periods, respectively, with expected values according to Equation 3:

(3) $$E\left(Y_{ja}\right)=\exp\left(\beta 2j-\lambda +\delta \right),\text{E}\left(Y_{jb}\right)=\exp\left(\beta 2j+\lambda -\delta \right),j=1,\ldots \text{nb}$$

Here, β1*i* and β2*j*​ represent participant-specific effects, λ represents the period or sequence-related effect, and δ represents the treatment effect. The null hypothesis of no difference in adverse-event rates between active and placebo IVR use was evaluated using an exact conditional test based on the Poisson crossover model. Conditioning on participant-specific totals and the relevant aggregate marginal total eliminated the participant-specific and period-effect nuisance parameters under the null hypothesis.

## 3 Results

### 3.1 Study participants

Trial participants provided informed consent and were screened as summarized in **Supplementary Figure 1**. A total of 15 healthy volunteers were screened, and 14 enrolled into PrEP-Pod-IVR-02 (**Supplementary Figure 1**). Of the 14 enrolled participants, 3 dropped out after completing Stage 1 (baseline, Visits 1-3, **Figure 1**) and 1 participant was withdrawn after Visit 2. Data from the 4 dropouts were not included in the analyses. Ten participants completed Stage 2 and 3 receiving both the active and placebo IVRs in a randomized sequence (5 participants per randomized group) for an exposure of 28 days per IVR (**Table 2**). There were no missed study visits, and three participants had an additional visit (ID 131-01, baseline; ID 131-02, Stage 3; and ID 131-08, Stage 3) for evaluation of AEs (see **3.2.** below).

**Table 2:** Demographics of the study participants who completed the PreP-Pod-IVR-02 trial.

Characteristic	
**Participants receiving both study products, *n* (percent)**	10 (100)
**Age (years), median (range)**	40 (29-44)
**Education (years)**	3 Associate’s degree; 3 Bachelor’s degree; 3 attended some college, 1 Graduate or professional degree
Partner status	
Living with partner	7 (1 main partner in sexual relationship)
Not living with partner	3 (1 reported having 1 main partner, but not in sexual relationship with this partner; 2 reported having 1 main partner and in sexual relationship with partner)
No partner	0
Race and Ethnicity, *n* (percent)	
Black or African American	2 (20)
White	
Hispanic	2 (20)
Non-Hispanic	6 (60)
Asian	0
Other	0
Contraceptive method used on study	
Hormonal methods^1^	12 at screening; 8 at completion (6 pills, 2 IUDs); 4 drop-outs (3 implants, 1 IUD)
Intrafallopian device	1
Sterilization of participant	1
Sterilization of partner	3
Abstinence	0
Consistent condom use	0
Tobacco use, *n* (percent)	3 (30)
Alcohol use, *n* (percent)	8 (80)
BMI^2^ (kg m^-2^), median (range)	29.5 (21.4-40.6)

^1^ Includes 3 implants, 3 IUDs, and 6 pills at screening, but no IVRs or DMPA; 2 participants used both male and female methods; 1 participant experienced expulsion of IUD at the time of Stage 2 IVR removal and used pills for the remainder of the study.

^2^ BMI, body mass index

### 3.2 Adverse events

All AEs, regardless of relationship to study product, are broken down in **Supplementary Table 1**, along with their Medical Dictionary for Regulatory Activities (MedDRA) categorization in **Supplementary Table 2**. There were 19 AEs related to IVR use (**Table 3**); 15 were Grade 1 AEs (79%) and 4 Grade 2 (21%) findings. These included vaginal discharge, vulvovaginal candidiasis, vulvovaginitis, bacterial vaginosis, menorrhagia, metrorrhagia, IUD expulsion, pollakiuria, lymphadenopathy, and abdominal pain. There were no SAEs or Grade 3 or 4 genitourinary AEs. The corresponding MedDRA categorization is presented in **Supplementary Table 3**.

**Table 3:** Related adverse events (AEs) during IVR use.

		Stage 1	Stage 2 & 3		Stage 2		Stage 3		Stage 2 & 3	
		Pre-Randomization (Baseline; No IVR) *N* = 14^1^	Active *versus* Placebo (crossover) *N* = 10		(1st IVR) *N* = 10; *N* = 5/arm		(2nd IVR) *N* = 10; *N* = 5/arm		per Arm (crossover) *N* = 10; *N* = 5/arm	
			Placebo	Active	Arm 1 Active	Arm 2 Placebo	Arm 1 Placebo	Arm 2 Active	Arm 1	Arm 2
**Total AEs**	**19**	**N/A**	**8**	**11**	**3**	**5**	**3**	**8**	**6**	**13**
Total Gr 1	15	N/A	6	9	1	4	2	8	3	12
Total Gr 2	4	N/A	2	2	2	1	1	0	3	1
Total Gr 3	0	N/A	0	0	0	0	0	0	0	0
Total Gr 4	0	N/A	0	0	0	0	0	0	0	0

^1^ Includes all participants eligible after screening visit.

Grade 1 AEs are mild and cause no or minimal interference with activity. Grade 2 AEs are controlled with medication or cause greater than minimal interference with activity.

Adverse event occurrences were episodic in nature, and the number of events within a time period generally followed a Poisson distribution (Layard and Arvesen, 1978). There was no statistically significant evidence of a treatment effect comparing adverse event counts during active *versus* placebo IVR use (H0​:δ = 0; *P* > 0.99; **Table 4**). There also was no statistically significant evidence of an order or period effect comparing related adverse event counts between Stage 2 and Stage 3 (H0​:λ = 0; *P* > 0.999).

**Table 4:** Fisher-Irwin exact tests of related AE occurrences during IVR use.

	Comparison related AEs by Tx (active *versus* placebo IVRs)			Comparison related AEs by arm (Tx order Stages 2 and 3 only)		
	Placebo IVR	Active IVR	Total by sequence	Stage 2	Stage 3	Total by sequence
**Sequence A/B**	x.a = 5	x.b = 8	x.. = 13	x.a = 5	x.b = 8	x.. = 13
**Sequence B/A**	y.a = 3	y.b = 3	y.. = 6	y.b = 3	y.a = 3	y.. = 6
**Total by comparison**	x.a + y.a = 8	x.b + y.b = 11	x.. + y.. = 19	x.. = 8	y.. = 11	x.. + y.. = 19
	**H0: δ = 0**			**H0: λ = 0**		
	***P* > 0.99**	No treatment effect detected		***P* > 0.99**	No order effect detected	

The table summarizes exact tests for related AE counts during the crossover portion of the trial. A denotes the placebo IVR and B denotes the active IVR. Participants in the *x* sequence received A followed by B, whereas participants in the *y* sequence received B followed by A. The treatment-effect test evaluates H0:δ = 0, corresponding to no difference in related AE counts between active and placebo IVR use. The period-effect test evaluates H0:λ = 0, corresponding to no difference in related AE counts between Stage 2 and Stage 3, independent of treatment assignment. Exact two-sided tests showed no statistically significant evidence of either a treatment effect or period effect.

### 3.3 Colposcopy and histology

There were a total of 34 colposcopy findings, and none were deemed related to IVR use. Most were common findings in women and of no clinical significance, such as ectopy and Nabothian cysts. During Stage 1 (baseline, **Figure 1**), one participant had papules related to a suspected allergy to swab collection materials and was withdrawn prior to Stage 2. Another participant had vaginal ulceration related to an HSV outbreak that was reported as an AE, treated, and the participant proceeded on study. There was one incidence of bleeding caused by the speculum (Stage 2, first IVR use, **Figure 1**). During Stage 3 (second IVR use, **Figure 1**) there were several instances of dark blood at the external cervical os, deemed to be menstrual blood. In one case, a fibrinous exudate was observed at a previous vaginal biopsy site.

Blinded histological examination of anterior mid-vaginal biopsies collected at Visits 7 and 12 (removal of the first and second IVRs, respectively, **Figure 1**) and Visit 13 (1–2 weeks post second IVR removal) did not identify significant differences between the active and placebo IVR groups in terms of the number of participants with histology events.

### 3.4 Optical coherence tomography

Optical coherence tomography (OCT) has been shown to provide a sensitive, noninvasive measure of changes in the vaginal and cervical epithelium thickness in a clinical setting (Vincent et al., 2011). The epithelial thickness of the vagina, fornix, and cervix as a function of IVR use is shown for the 10 trial participants in **Figure 2**.

**Figure 2:**
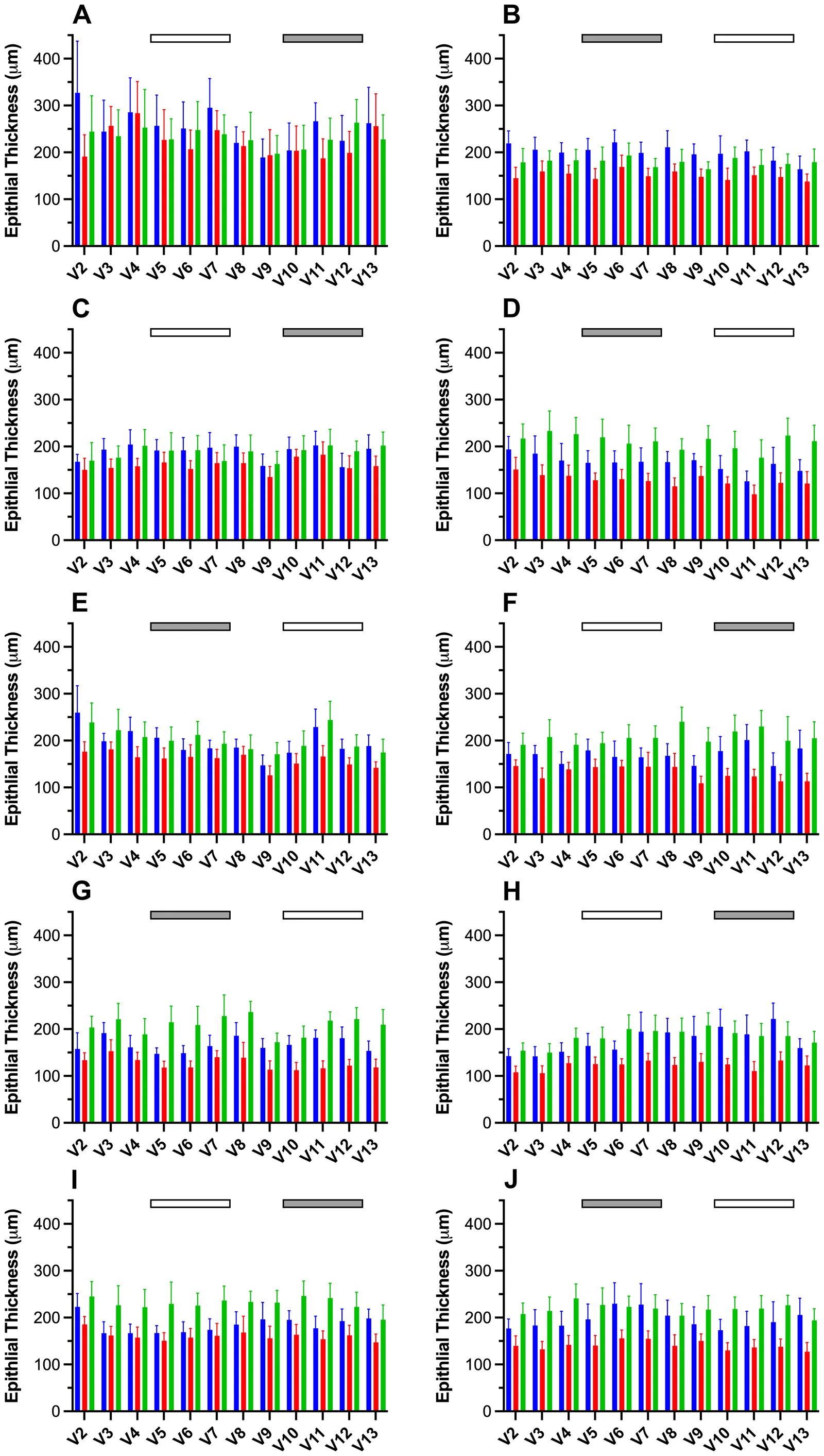
No systematic alteration in epithelial thickness (blue, vagina; red, fornix; green, cervix; shown as means + *SD*) of trial participants was observed during TDF-FTC IVR use. V2–13 Refer to clinical visits; white horizontal bars identify periods with placebo (unmedicated) IVR in place; grey horizonal bars identify periods with TDF-FTC IVR in place; **(A)** participant 131-02; **(B)** participant 131-05; **(C)** participant 131-06; **(D)** participant 131-07; **(E)** participant 131-08; **(F)** participant 131-09; **(G)** participant 131-11; **(H)** participant 131-12; **(I)** participant 131-13; **(J)** participant 131-15.

Epithelial thickness measured by OCT was analyzed separately for the vagina, fornix, and cervix using a crossover linear model with treatment condition and period as fixed effects and participant included to account for within-participant comparisons. Stage 1 baseline measurements were not included in the active-*versus*-placebo crossover comparison. No statistically significant differences in epithelial thickness were observed between active and placebo IVR periods for any compartment: vagina, active-placebo difference, 6.45 µm, *P* = 0.246; fornix, −1.98 µm, *P* = 0.683; and cervix, 2.87 µm, *P* = 0.489. In a previous study, we demonstrated that a decrease in epithelial thickness of 42 µm, or more, was significant in terms of local toxicity resulting from vaginal product (nonoxynol-9 gel) use (Vincent et al., 2011).

### 3.5 Vaginal pH and cell counts

Vaginal pH was variable across trial participants (**Supplementary Figure 2**), ranging between 3.6 and 6.1. For some women, the pH was stable throughout the duration of the trial (*e.g.*, 131-13, **Supplementary Figure 2I**), while for others it fluctuated widely (*e.g.*, 131-06, **Supplementary Figure 2C**). There was no clear association between IVR use and pH values or shifts.

Cell counts in vaginal swab, cervicovaginal lavage (CVL), and IVR surface samples on a per subject basis are shown in **Supplementary Figure 3**. The vaginal swab and CVL cell counts followed the same longitudinal trends. Overall, the envelope encompassing the range of vaginal swab cell counts was less variable and greater (*ca*. 10^6^–10^7^ cells) than for CVL samples (*ca*. 10^5^-5×10^6^ cells). For some women, however, the vaginal swab and CVL cell counts were comparable (*e.g.*, 131–02 and 131-12, **Supplementary Figures 3A, H**). The IVR surface cell counts could be binned into three groups: high (> 3×10^6^ cells), 131-06, 131-11; medium (3×10^5^-3×10^6^ cells), 131-02, 131-05, 131-07, 131-12, 131-13, 131-15; and low (< 3×10^5^ cells), 131-08, 131-09, (**Supplementary Figure 3**). The medium group had cell counts that typically were between the swab and CVL counts. There was no apparent association between IVR cell counts and vaginal microbiome (**Figure 3**).

**Figure 3:**
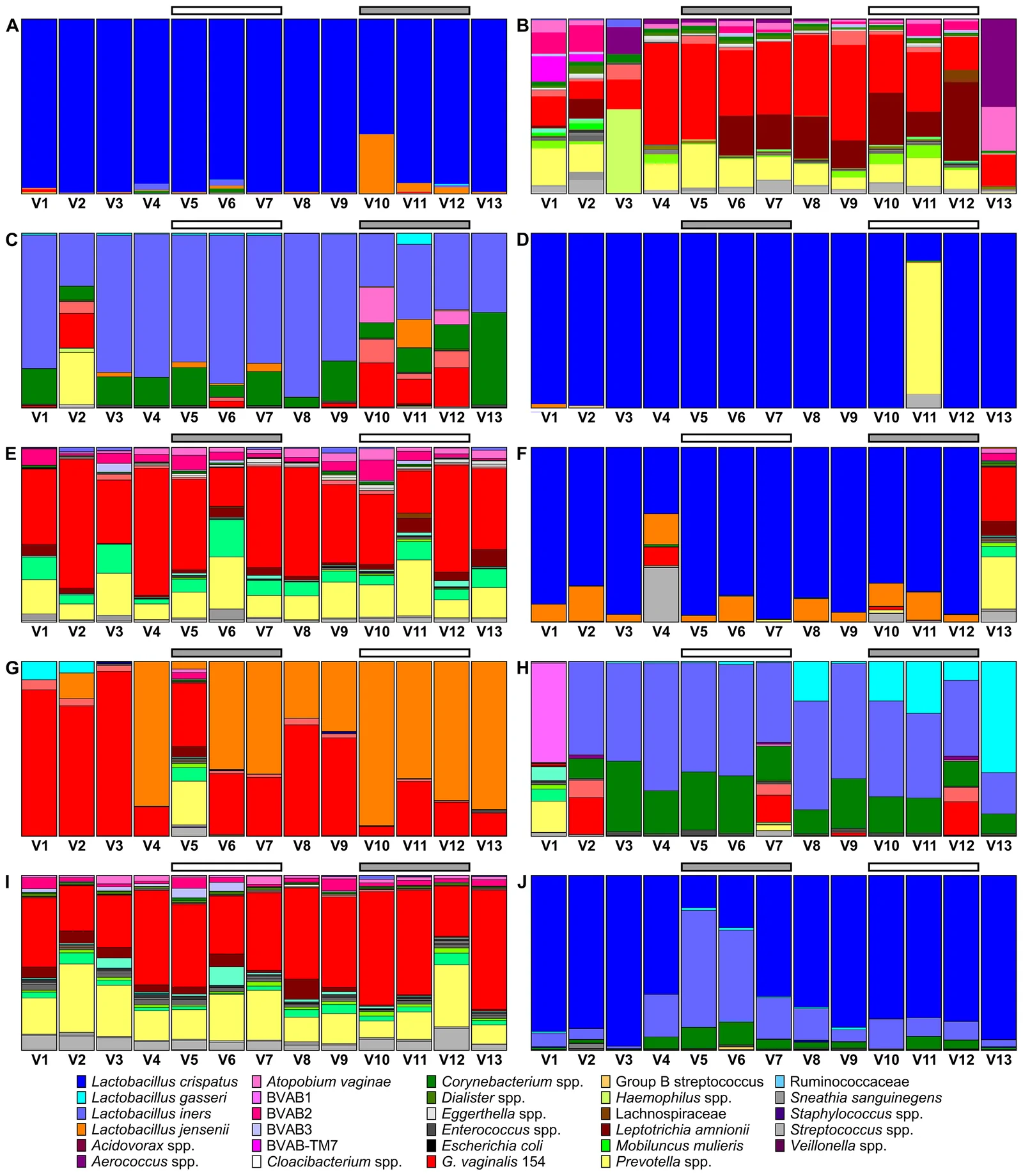
Vaginal microbiome composition of trial participants is not systematically altered in the presence of the TDF-FTC IVR. V1–13 Refer to clinical visits; white horizonal bars identify periods with placebo (unmedicated) IVR in place; grey horizonal bars identify periods with TDF-FTC IVR in place; **(A)** participant 131-02; **(B)** participant 131-05; **(C)** participant 131-06; **(D)** participant 131-07; **(E)** participant 131-08; **(F)** participant 131-09; **(G)** participant 131-11; **(H)** participant 131-12; **(I)** participant 131-13; **(J)** participant 131-15.

### 3.6 Vaginal microbiome community structure

The dynamics of the vaginal microbiome (VMB) in the presence of study product (placebo and active) relative to baseline are an important measure of local safety. The VMB structure for the 10 trial participants over all 13 clinical visits (V1-13), a total of 130 swabs analyzed, is shown in **Figure 3** and summarized in **Table 5**. Each panel indicates timeframes when either the placebo IVRs (white horizonal bars) or the TDF-FTC IVRs (grey horizonal bars) were in place.

**Table 5:** Summary of the most abundant vaginal microbiota at baseline and during IVR use for each study participant.

Participant	Baseline	Placebo D0	Placebo D7	Medicated D0	Medicated D7
131-02	*L. crispatus*	*L. crispatus*	*L. crispatus*	*L. crispatus*	*L. crispatus, L. jensenii*
131-05	Unstable, polymicrobial, anaerobic	*G. vaginalis*, *Leptotrichia* spp., *Prevotella* spp.	*G. vaginalis*, *Leptotrichia* spp., *Prevotella* spp.	*G. vaginalis*, *Prevotella* spp.	*G. vaginalis*, *Leptotrichia* spp., *Prevotella* spp.
131-06	*L. iners*, *Corynebacterium* spp.^1^	*L. iners*, *Corynebacterium* spp.	*L. iners*, *Corynebacterium* spp.	*L. iners*, *Corynebacterium* spp.	*L. iners*, *Corynebacterium* spp., *G. vaginalis*, *A. vaginae*
131-07	*L. crispatus*	*L. crispatus*	*L. crispatus^2^*	*L. crispatus*	*L. crispatus*
131-08	Unstable, polymicrobial, anaerobic	*G. vaginalis*, *Prevotella* spp.	*G. vaginalis*, *Prevotella* spp.	*G. vaginalis*, *Prevotella* spp.	*G. vaginalis*, *Prevotella* spp., incl. *P.* G1
131-09	*L. crispatus, L. jensenii*	*L. crispatus, L. jensenii, Streptococcus* spp., *G. vaginalis*	*L. crispatus, L. jensenii*	*L. crispatus, L. jensenii*	*L. crispatus, L. jensenii*
131-11	*G. vaginalis*	*G. vaginalis, L. jensenii*	*L. jensenii*	*G. vaginalis, L. jensenii*	Unstable, polymicrobial, anaerobic^3^
131-12	Unstable, polymicrobial, anaerobic;^1^ *L. iners*, *Corynebacterium* spp.	*L. iners*, *Corynebacterium* spp.	*L. iners*, *Corynebacterium* spp.	*L. iners*, *Corynebacterium* spp.	*L. iners*, *L. gasseri*, *Corynebacterium* spp.
131-13	*G. vaginalis*, *Prevotella* spp.	*G. vaginalis*, *Prevotella* spp.	*G. vaginalis*, *Prevotella* spp.	*G. vaginalis*, *Prevotella* spp.	*G. vaginalis*, *Prevotella* spp.
131-15	*L. crispatus*, *L. iners* ^4^	*L. crispatus, L. iners*, *Corynebacterium* spp.	*L. crispatus, L. iners*	*L. crispatus, L. iners*, *Corynebacterium* spp.	*L. crispatus, L. iners*, *Corynebacterium* spp.

In half of the cases, the medicated IVR preceded the placebo IVR because of randomization. *A. vaginae*, *Atopobium vaginae*; *G. vaginalis*, *Gardnerella vaginalis*; *L. crispatus*, *Lactobacillus crispatus*; *L. gasseri*, *Lactobacillus gasseri*; *L. iners*, *Lactobacillus iners*; *L. jensenii*, *Lactobacillus jensenii*.

^1^ Short period (Visit 2) of unstable, polymicrobial, anaerobic vaginal microbiota during baseline; resolved by Visit 3.

^2^ Vaginal microbiome dominated by *Prevotella* spp. on D14, along with *Streptococcus* spp. and *L. crispatus; back to L. crispatus dominant by D21*

^3^ Back to *G. vaginalis*, *L. jensenii* dominant by D14 (Visit 6)

^4^ *Corynebacterium* spp. at Visit 4

Over the course of the trial, 7 out of 10 participants had VMBs that were *Lactobacillus*-rich. The VMB of 4 participants was dominated by *Lactobacillus crispatus* (**Figures 3A, D, F, J**). The VMB of 2 participants consisted predominantly of *L. iners* (**Figures 3C, H**), while the VMB of another participant consisted primarily of *L. jensenii* (**Figure 3G**). The VMBs of the remaining 3 participants were rich in *Gardnerella vaginalis* and other organisms associated with bacterial vaginosis profiles (**Figures 3B, E, I**). 

No reproducible shifts associated with either placebo or active IVR use were observed. The baseline sampling period (*i.e.*, prior to IVR placement) aided in contextualizing the VMB variability while evaluating IVR impacts. During the course of the trial, 4 participants experienced shifts in some bacterial populations that were not consistently correlated with either IVR, but in some cases were associated with transient vaginal fungal infections. Specifically, participant 131–05 had fungal PCR positive samples on V2, 3, 7 and 10 with subsequent expansion of *Leptotrichia* (**Figure 3B**, dark red) at V6-V12 primarily when the placebo IVR was in place. Participant 131–06 experienced a PCR-detected fungal infection (V9, 10 and 12) coincident with TDF-FTC IVR use, and a perturbation in the otherwise stable *L. iners*-dominated VMB (**Figure 3C**). Participant 131–11 experienced an expansion of *L. jensenii* after the medicated IVR was used that was maintained with the placebo IVR was in place, but had no fungal detections (**Figure 3G**). Finally, participant 131–15 experienced a *L. iners* bloom with the medicated IVR in place that became the dominant bacterial species on V5 followed by a return to *L. crispatus* dominance by V7 (**Figure 3J**). These changes also were associated with fungal detections (V1, 2, 3 and 5). Collectively, the PCR findings support the conclusion that neither the placebo nor active IVRs consistently altered the VMB.

### 3.7 *In vivo* ARV drug release rates

Over 28 d of use, the IVRs released 24.3 ± 18.6 mg TDF and 28.2 ± 13.3 mg FTC, corresponding to *in vivo* release rates of 0.87 ± 0.67 and 1.01 ± 0.48 mg d^-1^, respectively, assuming zero order kinetics. Analysis by HPLC showed that, 96.6 ± 0.5% (range 96.2 - 97.6%) of the residual TDF in the used IVR pods consisted of the hydrolytically labile TDF prodrug; *i.e.*, minor degradation to the metabolites (see **Figure 4**) following one month of use *in vivo*.

**Figure 4:**
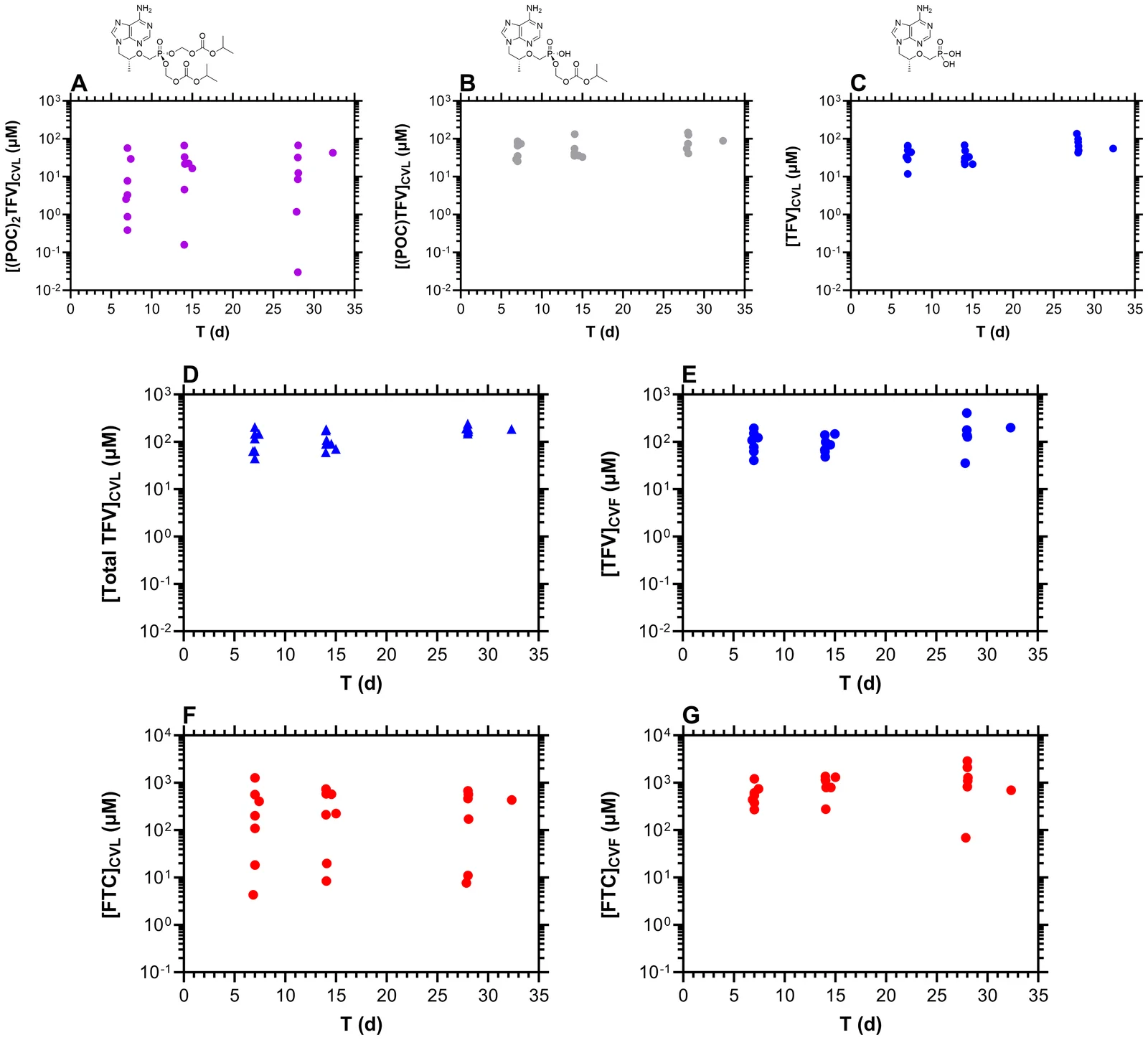
Cervicovaginal lavage drug and drug metabolite concentration-time profiles, all corrected for CVL dilution, agree favorably with the corresponding swab-collected CVF values. Every circular and triangular datum represents an individual, quantifiable sample from one of the participants. **(A)** TFV disoproxil in CVL. **(B)** TFV monoisoproxil in CVL. **(C)** TFV in CVL. **(D)** Molar sum of all three TFV species in CVL. **(E)** TFV in swab-collected CVF. **(F)** FTC in CVL. **(G)** FTC in CVF.

### 3.8 Cervicovaginal fluid drug-concentration time profiles

Drug and drug metabolite concentrations in key anatomic compartments with the IVRs in place are summarized in **Table 6**. These aggregated data are supported by the horizontal analyte concentration-time profiles with the active IVRs in place (**Figure 5**). Median, molar CVF TFV concentrations were 7 times lower than median FTC concentrations.

**Table 6:** Summary of antiretroviral (ARV) drug concentrations in key anatomic compartments with the TDF-FTC IVR in place (Visits 5-7/10-12).

Analyte, matrix, concentration units	*n*	% > LLQ^1^	Median (IQR)^2^
Cervicovaginal fluid, TFV	30	100	
fmol mg^-1^			136×10^3^ (86.8×10^3^-186×10^3^)
ng mg^-1^			39.1 (24.9-53.4)
Cervicovaginal fluid, FTC	30	100	
fmol mg^-1^			984×10^3^ (620×10^3^-1, 307×10^3^)
ng mg^-1^			243 (153-323)
Vaginal tissue, TFV	10	100	
fmol mg^-1^			10.9×10^3^ (4.90×10^3^-16.0×10^3^)
ng mg^-1^			3.14 (1.41-4.60)
Vaginal tissue, TFV-DP	10	100	
fmol mg^-1^			0.651×10^3^ (0.307×10^3^-1.40×10^3^)
Vaginal tissue, FTC	10	100	
fmol mg^-1^			15.8×10^3^ (13.2×10^3^-22.4×10^3^)
ng mg^-1^			3.89 (3.26-5.55)
Vaginal tissue, FTC-TP	10	90	
fmol mg^-1^			175 (104-198)
Rectal tissue, ^3^ TFV	20	20	
fmol mg^-1^			*225, 711, 458, 374*
ng mg^-1^			*0.0647, 0.204, 0.132, 0.107*
Rectal tissue, ^3^ TFV-DP	20	15	
fmol mg^-1^			*3.60, 2.40, 2.39*
Rectal tissue, ^3^ FTC	20	5	
fmol mg^-1^			*4.99×10^3^*
ng mg^-1^			*1.23*
Rectal tissue, ^3^ FTC-TP	20	0	
Rectal fluid, TFV	10	70	
fmol mg^-1^			650 (129-1.57×10^3^)
ng mg^-1^			0.187 (0.0370-0.452)
Rectal fluid, FTC	10	70	
fmol mg^-1^			2.24×10^3^ (391-3.46×10^3^)
ng mg^-1^			0.554 (0.0967-0.855)
Plasma, TFV	30	3.2	
ng mL^-1^			*0.48*
Plasma, FTC	30	68	
ng mL^-1^			0.539 (0.157-0.757)

Commonly used units (*e.g.*, ng mg^-1^ for cervicovaginal fluids) are presented for key compartments as well as molar units to allow meaningful comparison. Analyses that were BLQ were treated according to eq. 1. For groups where less than 25% of samples were quantifiable, individual measurements are presented in italics.

^1^ Proportion of samples with quantifiable drug concentrations.

^2^ Interquartile range (25th to 75th percentile)

^3^ Anterior and posterior rectal biopsies were collected and analyzed

**Figure 5:**
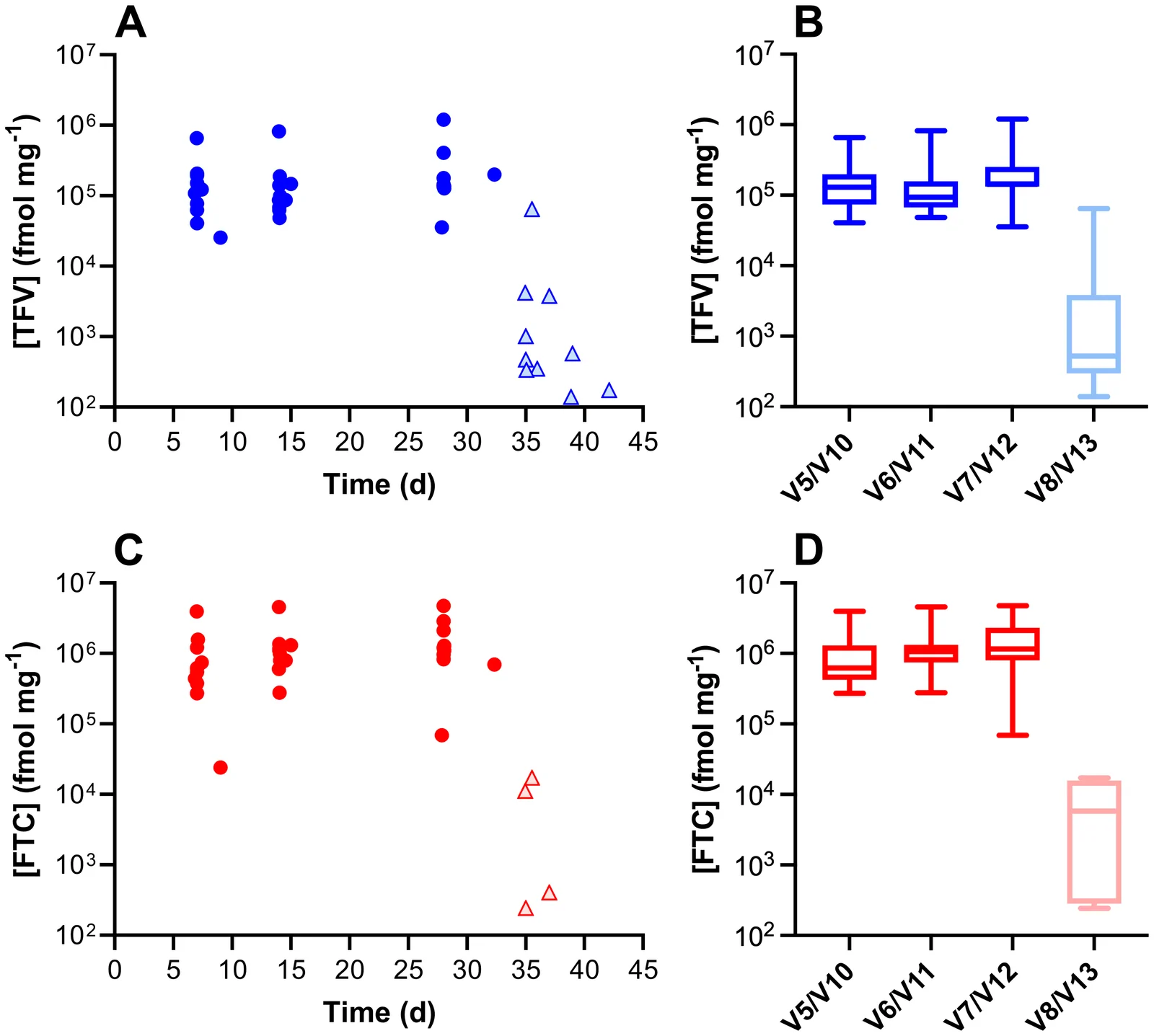
Cervicovaginal fluid, swab-collected, drug and drug metabolite concentrations and washout in the TDF-FTC IVR group. Dark blue circles and box plots, TFV concentrations with the IVR in place; pale blue triangles and box plots, TFV concentrations following IVR removal; Dark red circles and box plots, FTC concentrations with the IVR in place; pale red triangles and box plots, FTC concentrations following IVR removal. Every circular and triangular datum represents an individual, quantifiable sample from one of the participants and corresponds to **(A)** TFV and **(C)** FTC concentrations. The corresponding summary data as a function of study visit are shown as box plots for **(B)** TFV and **(D)** FTC. The box extends from the 25th to 75th percentiles, with the horizontal line in the box representing the median; whiskers represent the lowest and highest datum. The median mass of CVF collected per swab was 60 mg. 1 fmol mg^-1^(TFV) = 0.29 pg mg^-1^; 1 fmol mg^-1^(FTC) = 0.25 pg mg^-1^. Samples that were BLQ were omitted from the graphs.

The cervicovaginal fluid (swab-collected) drug and drug metabolite concentrations resulting from TDF-FTC IVR use are shown in **Figure 5** in two formats: (1) individual concentration-time profiles, with the pale triangles representing the measurements following IVR removal, *i.e.*, washout, (TFV, **Figure 5A**; FTC, **Figure 5C**); (2) box plots representing summary concentration data as a function of study visit, with the IVRs removed at Visit 8 (Stage 2) and 12 (Stage 3). All drug analyses of vaginal swab samples from the placebo group were BLQ. The drug washout for TFV was slower than for FTC, in agreement with results from our 7-day TDF-FTC pod-IVR trial; median terminal half-lives: TFV, TFV, 31.4 h; FTC, 19.1 h (Vincent et al., 2018b).

Cervicovaginal lavage (CVL) samples were collected using lithium (as LiCl) as an exogenous tracer to enable calculation of the CVF dilution during the lavage procedure (Churchman et al., 2016). The distribution of corrected CVF mass collected by lavage in the trial is shown in **Figure 6**. Data from participants 131-12, -13, and -15 were omitted due to a small amount of sample evaporation during handling, thereby compromising the measurements.

**Figure 6:**
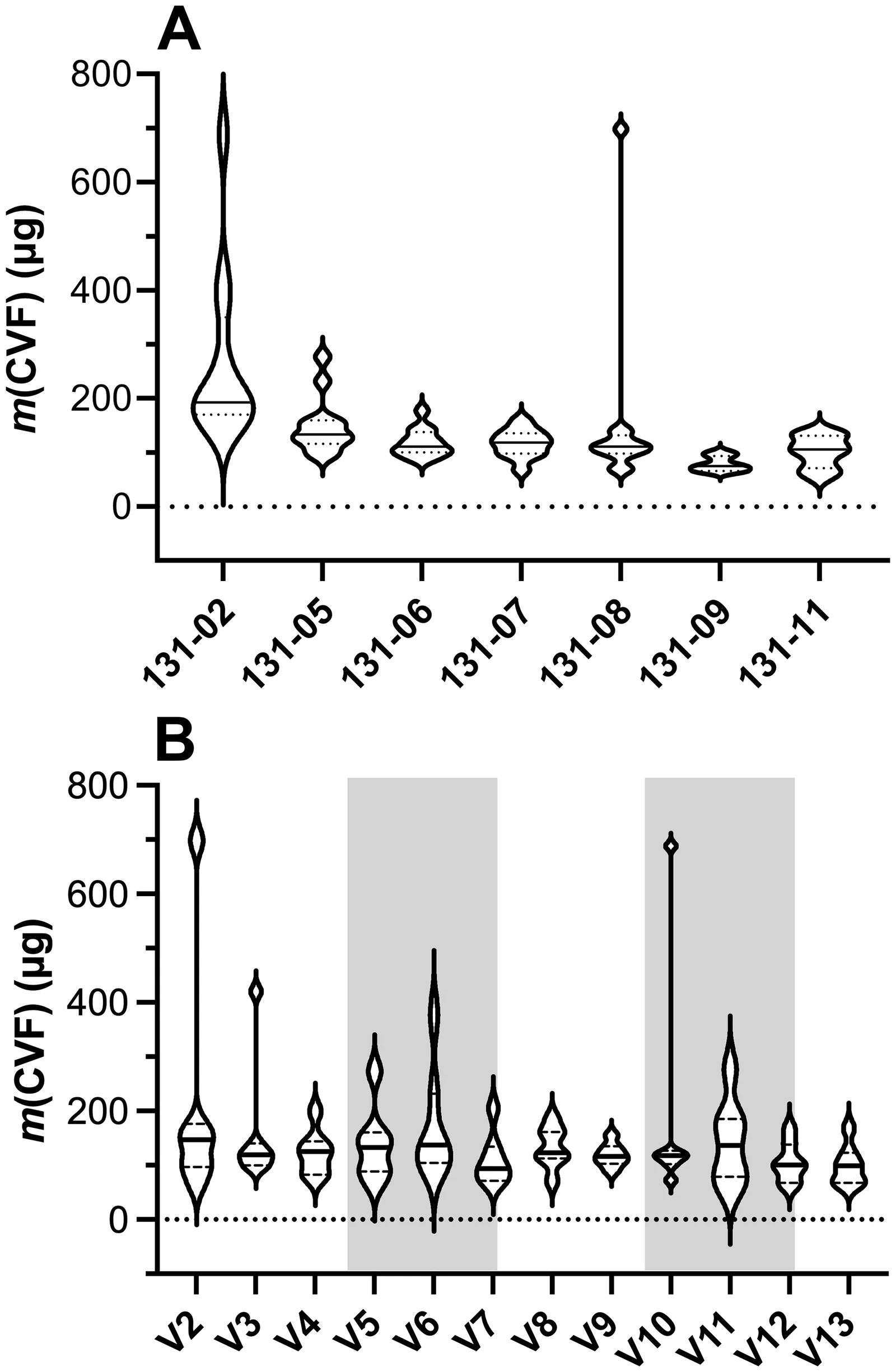
Violin plots showing the corrected mass of CVF collected by lavage during the trial. Solid horizonal line, median; dotted lines, quartiles. Data are shown as a function of **(A)** participant ID and **(B)** study visit, with the grey shaded boxes identifying periods of IVR use.

The median mass of CVF collected typically ranged between 0.1-0.2 mg across participants (**Figure 6A**) and study visits (**Figure 6B**) and did not appear to be related to IVR use. Since the CVL samples were collected after other vaginal specimens (5 vaginal swabs followed by 2 vaginal wall scrapings, see **Supplementary Material**), the naïve CVF mass (*i.e.*, original CVF mass prior to any sample collection) likely was much higher than the mass measured in the CVL.

Concentration-time plots summarizing CVF and CVL data are presented in **Figure 4**. Molar concentrations of TFV disoproxil [(POC_2_)TFV, **Figure 4A**], the intermediate TFV monoisoproxil (POC-TFV, **Figure 4B**), and TFV (**Figure 4C**), all corrected for dilution, illustrate the distribution of the administered agent (TDF) and the two metabolites. The hydrolytically labile (POC_2_)TFV was highly variable, while the two metabolites displayed equally stable concentration profiles. Corrected CVL total TFV concentrations (**Figure 4D**, molar sum of all three species shown in **Figures 4A–C**) compared favorably with the corresponding swab-collected CVF TFV concentrations (**Figure 4E**), while FTC CVL concentrations were more variable over time (**Figure 4F**). The overall agreement across CVF and CVL measurements, especially for TFV, is encouraging not only because of the different analytical approaches, but also because the analysis was performed in different laboratories.

The distribution of the three TFV species in CVL samples during TDF-FTC IVR use as a function of vaginal pH and microbiome is shown in **Figure 7**. The proportion of each analyte is represented as the dimensionless mole fraction, *χ*. For example, *χ(POC-TFV)* = [POC-TFV]/{[TDF]+[POC-TFV]+[TFV]}, on a molar basis. In *Lactobacillus crispatus*-dominated vaginal microbiomes, the mole fraction of all three analytes was comparable across study days. When the microbiome consisted mainly of *L. iners*, *χ(TFV)* was greater than the other two, indicating favorable conditions for complete hydrolysis of the two isoproxil prodrug groups (**Figure 7H**). In cases of high *Gardnerella vaginalis* representation, an accumulation of the POC-TFV was observed (**Figures 7B, E, I**).

**Figure 7:**
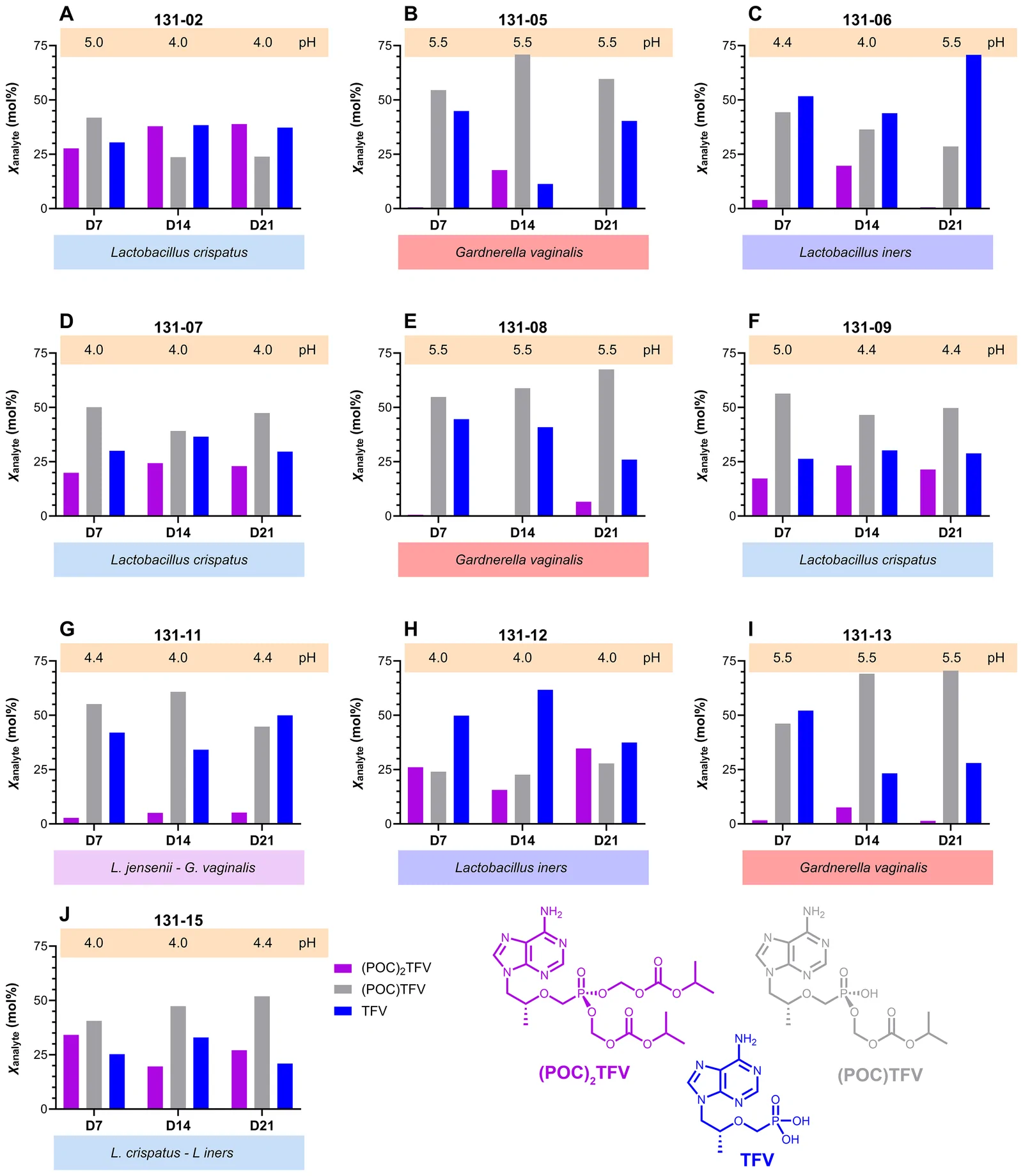
The mole fraction, *χ*, of TFV species in CVL samples is somewhat related to the predominant vaginal microbiota. D7, D14, and D21 represent days of IVR use when CVL samples were collected. The three chemical structures correspond to the figure legends by color; **(A)** participant 131-02; **(B)** participant 131-05; **(C)** participant 131-06; **(D)** participant 131-07; **(E)** participant 131-08; **(F)** participant 131-09; **(G)** participant 131-11; **(H)** participant 131-12; **(I)** participant 131-13; **(J)** participant 131-15.

Concentrations of TFV and FTC in CVF were compared across two groups by VMB composition (**Supplementary Figure 4**): *L. crispatus*-dominant (participant 131-02, -07, -09) and *G. vaginalis*-dominant (participant 131-05, -08, -13). Log-transformed TFV and FTC concentrations were analyzed using a repeated-measures model that included the VMB group and study visit as fixed effects. Statistical significance was assessed using an exact participant-level permutation test where each participant’s 3 repeated measurements were retained together. There was no evidence that *L. crispatus*–dominant microbiomes were associated with higher TFV or FTC concentrations. The visit-adjusted geometric-mean ratios comparing *L. crispatus*–dominant with *G. vaginalis*–dominant participants were 0.45 for TFV and 0.49 for FTC. For both outcomes, the exact one-sided *P*-value for higher concentrations in the *L. crispatus* group was 0.95.

### 3.9 Drug and drug metabolite concentrations in vaginal tissue and rectal fluid samples

Drug and drug metabolite concentrations on a molar basis in vaginal tissue (VT) biopsies collected during TDF-FTC IVR use are summarized in **Figure 8A** for direct comparison. Except for FTC-TP, all measurements were above the LOQ (**Table 6**). While the median FTC VT concentration was 1.4 times higher than the corresponding TFV concentration, the median TFV-DP concentration was 3.7 times greater than that of FTC-TP (**Table 6**).

**Figure 8:**
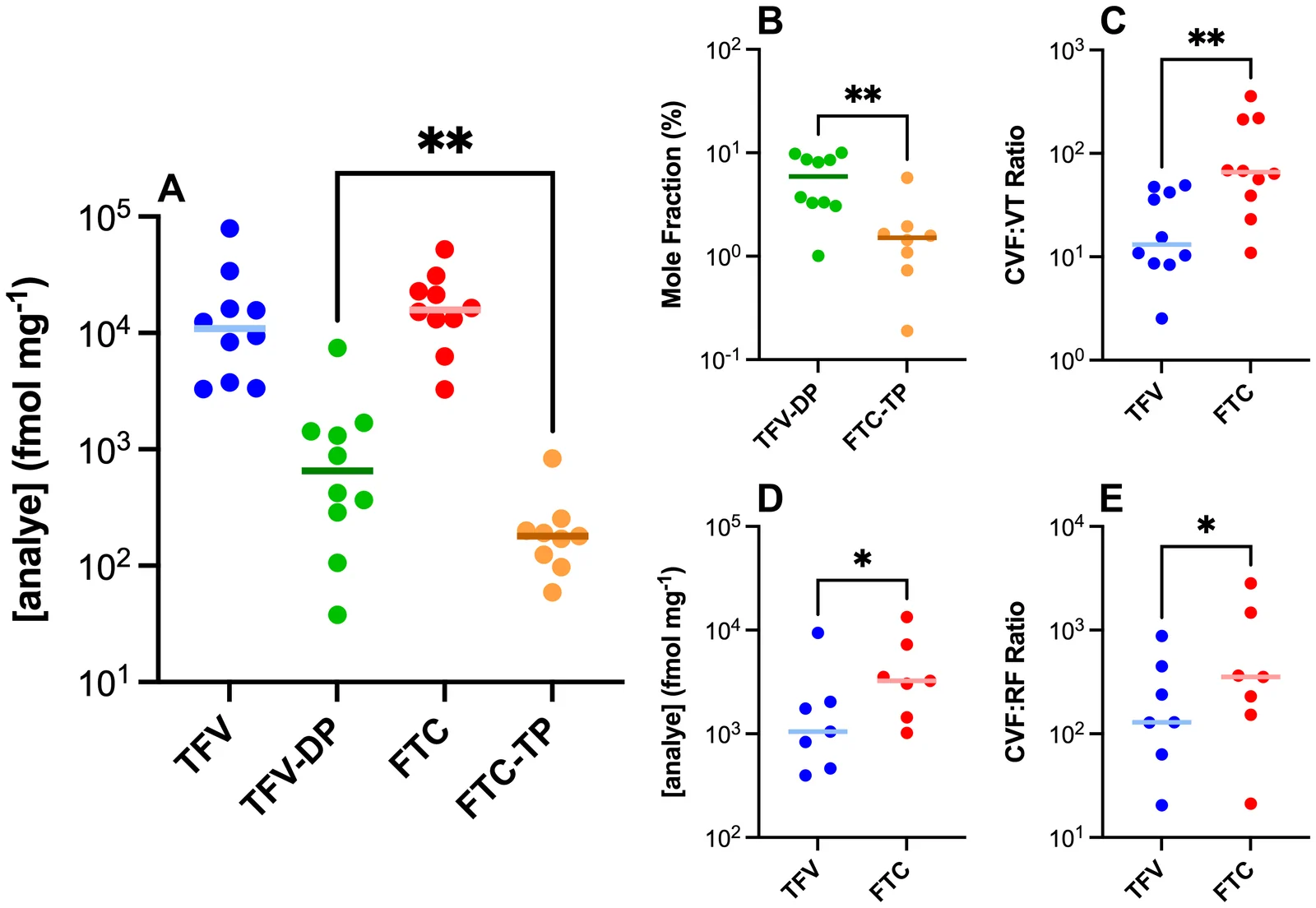
The concentrations of TFV, FTC, and their anabolites (where relevant), TFV-DP, FTC-TP, were quantifiable in most vaginal tissue (VT) and rectal fluid (RF) samples collected after 28 d of IVR use (Visit 7/13). Every circular and triangular datum represents an individual, quantifiable sample from one of the participants. **(A)** Molar vaginal tissue concentrations; **(B)** phosphorylated anabolite mole fractions in vaginal tissues; **(C)** cervicovaginal fluid-to-vaginal tissue concentration ratios; **(D)** molar rectal fluid concentrations; **(E)** cervicovaginal fluid-to-rectal fluid concentration ratios. Paired measurements were compared using the Wilcoxon matched-pairs signed-rank test; *P ≤ 0.05; **P ≤ 0.01. Samples that were BLQ were omitted from the graphs.

Paired datasets shown in **Figure 8** were compared using a nonparametric Wilcoxon matched-pairs signed rank test. While the CVF molar TFV and FTC concentrations differed by a factor of 7, the corresponding VT concentrations (**Figure 8A**) were not significantly different (*P* = 0.3223). The matched VT anabolite concentrations (TFV-DP and FTC-TP) were significantly different (*P* = 0.0078, **Figure 8A**). The VT TFV phosphorylation efficiency, assessed by median VT *χ(TFV-DP)*, was greater than FTC phosphorylation efficiency, *χ(FTC-TP)*, 5.9 and 1.5%, respectively (*P* = 0.0078, **Figure 8B**).

While less than 10% of samples had concentrations of measured analytes above the LOQ in rectal tissue samples, 70% of rectal fluid samples were above the analytical LOQ for TFV and FTC (**Table 6**). Median FTC rectal fluid concentrations were 3 times higher than TFV (*P* = 0.0156, **Figure 8D**).

Paired concentration ratios spanning anatomic compartments provide a measure of drug distribution. The median of individual cervicovaginal fluid-vaginal tissue (CVF: VT) ratios (**Figure 8C**) for TFV and FTC were 13 and 68, respectively, a 5-fold difference (*P* = 0.0059). The median CVF-rectal fluid (CVF: RF) ratios (**Figure 8E**) for TFV and FTC were 129 and 354, respectively, a 3-fold difference (*P* = 0.0469).

## 4 Discussion

Our placebo-controlled, randomized, crossover trial found that the pod-IVR generally was well-tolerated and safely delivered the TDF-FTC combination in healthy women over 28 days of consecutive use. These findings are consistent with our pilot 7-day trial (Guthrie et al., 2018; Vincent et al., 2018a, 2018). All 10 participants completed the 3 trial stages, despite challenges associated with conducting the trial during the COVID-19 pandemic. The blinded crossover design helped minimize bias due to order of administration for all safety measures, and the Stage 1 baseline period was informative at characterizing trends and variability prior to using the IVRs. Despite the utility of Stage 1, the randomized active-*versus*-placebo IVR comparison (Stages 2 and 3) was the primary analysis (*i.e.*, not compared statistically with baseline as a third randomized condition) because it preserves the crossover design and controls for between-participant variability. The baseline period was not randomized and differed from the treatment periods both temporally and because no IVR was present. There were no concerning drug product-related AEs. There was no statistically significant evidence of a difference in related AE counts between active and placebo IVR use or between study periods; however, because only 10 participants completed both treatment periods and 19 related adverse events were observed, these analyses had limited precision and cannot exclude clinically meaningful treatment or period effects.

Optical coherence tomography (OCT) is a sensitive and quantitative technique for measuring changes in vaginal epithelium thickness *in vivo* that has been used preclinically and clinically to evaluate vaginal products (Vincent et al., 2008, 2009, 2011, 2013). Here, OCT was used to intensely monitor epithelial changes of the vaginal, forniceal, and cervical compartments and no statistically significant differences between the active and placebo groups were found. We also measured no systematic or consistent alterations of the VMB community structures as a function of IVR use, in agreement with our previous pod-IVR trials (Keller et al., 2012; Ursell et al., 2014; Vincent et al., 2018b).

Intravaginal rings typically maintain low (compared with gels and inserts), constant CVF drug concentrations that lead to low systemic drug absorption. Most of the plasma TFV measurements with the active IVRs in place were BLQ, and the median FTC concentration was 0.54 ng mL^-1^, likely explaining the lack of any observed systemic AEs. For context, trough concentrations of these two analytes with oral daily dosing of TDF-FTC are far higher, reported as 52 ng mL^-1^ and 71 ng mL^-1^, respectively, in a month-long directly observed dosing study (Hendrix et al., 2016).

Keller et al. evaluated a polyurethane (PU) reservoir-type IVR delivering TDF in 12 participants, but the trial was ended early due to safety concerns related to product use (Keller et al., 2019). Eight participants developed grade 1 genital ulcers with a mean 32 d of IVR use (range 23–56 d). Median TFV CVF concentrations on Day 14 for the above 8 participants were 1.0×10^5^ (IQR 5.6×10^4^- 1.1×10^5^) ng mL^-1^, but became more variable as the study progressed. By Day 28, the median TFV CVF concentrations in the participants who developed ulcers were 6·0×10^4^ (IQR 3·2×10^4^-9·6×10^4^) ng mL^-1^. These values are 2.6× (Day 14) and 1.5× (Day 28) higher than observed here. The vaginal tissue TFV-DP concentrations measured in our study were *ca*. double those measured in ectocervical tissue on Day 28 by Keller et al. Based on the PKs of the TDF-FTC and PU TDF IVRs, it seems unlikely that the vaginal drug concentrations alone account for the different safety profiles of the devices. One possible explanation for the vaginal ulceration is related to the IVR material, construction (the IVRs consisted of drug-containing hydrophilic PU tubing sealed by induction-melt welding) (Smith et al., 2013), and mechanical properties, in combination with the TDF metabolites. Our pod-IVRs consist of a softer material that is silicone injection-molded into a torus, thereby avoiding the formation of a potentially irritating weld joint.

The IVRs maintained robust and stable TFV and FTC CVF concentrations with the devices in place, followed by rapid washout once they were removed. The hydrolytically unstable TDF prodrug did not degrade in the pods following 28 days of IVR use. As in our pilot trial, the TFV washout was slower than FTC (Vincent et al., 2018b). Cervicovaginal fluid TFV and FTC concentrations were measured using two different sampling approaches: swab-collected and weighed; and CVL-collected and corrected for dilution using an exogenous tracer (Churchman et al., 2016). Both methods led to similar concentration-time profiles for TFV, but the FTC measurements were more variable in CVL samples, biased by 2 outliers on Days 7 and 14, and 1 on Day 28 (**Figure 4**). The cause for the differences is unclear as the CVL dilution factor for both analytes should be the same.

While CVF TDF concentrations were low and highly variable during active IVR use, likely due to esterase-mediated hydrolysis, the intermediate metabolite POC-TFV molar concentrations were comparable to TFV. This constitutes an important PK finding as POC-TFV is not normally analyzed and may lead to an underestimation of the concentration of TFV species in CVF. Interestingly, an association between TFV species (TDF, POC-TFV, TFV) mole fractions and the dominant vaginal microbiota was observed. In *L. crispatus*-dominated VMBs, the mole fractions of the 3 species were similar, with occasionally higher POC-TFV values.; however, *L. iners*-rich VMBs increased the TFV mole fractions, while reducing the TDF values. The vaginal pH of these samples, dominated by either *L. crispatus* or *L. iners*, was similar. *G. vaginalis*-dominant VMBs diminished the TDF mole fractions and generally were highly represented by POC-TFV. Ours is the first report of VMB-dependent metabolic profiles of TFV species following vaginal dosing. The implications of these findings are unclear and merit further clinical investigation in a larger population.

A retrospective, subset analysis of the CARPISA 004 Phase 2b clinical trial of pericoitally dosed TFV 1% vaginal gel (Abdool Karim et al., 2010) found that product efficacy at preventing HIV-1 infection was related to VMB composition (Klatt et al., 2017). Tenofovir was 61% (*P* = 0.0013) effective in women with *Lactobacillus*-dominant VMBs, but only 18% (*P* = 0.644) in women with *G. vaginalis*-rich VMBs, along with other anaerobes. Metabolism of TFV to adenine by *G. vaginalis*, but not Lactobacilli was suggested as a partial explanation. Although not statistically significant, we found that median TFV and FTC CVF concentrations during active IVR use were consistently higher in *G*. *vaginalis*-dominant compared with *L. crispatus*-rich VMBs. The sparse nature of our datasets (3 participants per group) prevents conclusions to be drawn from our results regarding bacterial metabolism of TFV and FTC to adenine and cytosine, respectively. The role of the VMB in determining ARV drug disposition likely is more complex, as suggested by other studies (Taneva et al., 2018), and dependent on the nature of the agent (*e.g.*, TDF *versus* TFV) as well as the mode of topical administration (*e.g.*, IVR *versus* pericoital gel).

Vaginal delivery of TDF *via* IVR leads to significantly greater TFV tissue penetration compared with FTC as reported previously (Vincent et al., 2018b). The difference also was reflected in significantly higher molar TFV-DP vaginal tissue concentrations than FTC-TP, the anabolites primarily responsible for inhibiting HIV-1 reverse transcriptase. Note that FTC-TP was not measured in vaginal tissues in our pilot clinical trial. Median TFV-DP vaginal tissue concentrations were 2.3× higher than in our 7-day pilot trial.

In our 7-day trial, we unexpectedly detected measurable TFV and FTC in rectal fluid samples, with median concentrations, 1.1 and 20 ng mg^-1^, respectively (Vincent et al., 2018a). The corresponding drug concentrations measured in the current trial were similar (TFV) or lower (FTC). The rectal tissue drug concentrations, not collected in the pilot trial, were mostly BLQ. We collected paired anterior (proximal to the vagina) and posterior (distal to the vagina) rectal biopsies. If passive drug diffusion occurred directly from the vaginal to the rectal tissues, and then into the rectal fluids, we should have observed consistently higher anterior rectal tissue concentrations. These data suggest that rectal drug exposure following IVR delivery may result from vaginal leakage rather than diffusion through tissues between the two compartments, although the implication of membrane transporters and active flux though tissues cannot be ruled out.

There have been no clinical trials evaluating the efficacy of vaginally co-administered TDF and FTC, so meaningful extrapolation from our PK data is challenging. Secondary analysis of CAPRISA 004 (pericoital 1% TFV vaginal gel) suggested that CVF TFV concentrations exceeding 1,000 ng mL^-1^ (1 ng mg^-1^) were associated with 76% protection against HIV-1 acquisition (Karim et al., 2011; Kashuba et al., 2015), *ca*. 40× lower than the concentrations observed here during IVR use. Median CVF TFV and FTC concentrations measured with the active IVRs in place were comparable or lower to those in a previous pod-IVR study that achieved complete protection from SHIV162p3 infection in a rigorous, repeat low-dose vaginal exposure model using normally cycling female pigtailed macaques (TFV, 46 ng mg^-1^; FTC, 1.1×10^3^ ng mg^-1^) (Srinivasan et al., 2016). Vaginal dosing of TDF (32 µM) and FTC (0.6 µM) resulted in 100% protection from HIV-1 infection in humanized mice (Gallay et al., 2017), and higher concentrations (TFV, 140 µM; FTC, 970 µM) were maintained in the current trial during IVR use. Based on CVF drug concentrations and directly comparable results from two established efficacy animal models, the TDF-FTC pod-IVRs show encouraging pharmacodynamic potential.

In conclusion, we successfully completed an ambitious (14 visits), randomized order, placebo-controlled, double blind, crossover, Phase 1 trial involving 3 successive 28-day stages. The TDF-FTC IVRs safely delivered and maintained favorable drug concentrations in key anatomic compartments relevant for HIV-1 PrEP.

## Data Availability

The datasets presented in this study can be found in online repositories (Dataset DOI: 10.5061/dryad.c2fqz61s1) and in the supplementary material.
