# Supplementary material for: An intravaginal ring for HIV pre-exposure prophylaxis: assessment of safety and pharmacokinetics in a randomized, placebo-controlled clinical trial
Source: Front Cell Infect Microbiol. 2026 Oct 6;16:1955105. doi: 10.3389/fcimb.2026.1955105 (PMC13680163; doi:10.3389/fcimb.2026.1955105)
Supplement: Supplementary file 1 [file DataSheet1.pdf]

An Intravaginal Ring for HIV Pre-exposure Prophylaxis: Assessment of Safety and Pharmacokinetics in a Randomized, Placebo-controlled Clinical Trial

Supplementary Material

**SUPPLEMENTARY FIGURE 1.**	PrEP-Pod-IVR-02 clinical trial flow diagram, summarizing screening, enrollment, and volunteer participation
**SUPPLEMENTARY FIGURE 2.**	Vaginal pH of trial participants is not systematically altered in the presence of the TDF-FTC IVR
**SUPPLEMENTARY FIGURE 3**	Cell counts in vaginal samples collected from trial participants
**SUPPLEMENTARY FIGURE 4**	Cervicovaginal fluid (swab-collected) drug concentrations grouped by VMB
SUPPLEMENTARY TABLE 1	All AEs regardless of relationship to study product
SUPPLEMENTARY TABLE 2	MedDRA Categorization of all AEs regardless of relationship to study product
SUPPLEMENTARY TABLE 3	MedDRA Categorization of all AEs related to IVR use

Materials and Methods

The following clinical procedures and sample collections were performed:

- Pelvic exam (incl. colposcopy and optical coherence tomography),
- Pelvic ultrasound (transabdominal, translabial, and/or transvaginal ultrasound) to confirm IVR placement (V6 and V11),
- Vaginal swabs (*N* = 5) for PK (V4-13), pH, microbiology, multi-omics, cell counts *via* qPCR, and *ex vivo* pharmacodynamic studies,
- Vaginal wall scrapings (*N* = 2) for microbiology and multi-omics,
- Vaginal wall brushing (*N* = 1) for multi-omics,
- Cervicovaginal lavage (*ca*. 3 mL) for multi-omics and PK (V4-13),
- Cervicovaginal lavage (*ca*. 10 mL) for cytology, flow cytometry, and multi-omics, cell counts *via* qPCR,
- Plasma for PK (V4-14),
- Vaginal biopsies (*N* = 3) for PK, histology, and multi-omics (V7, V12, and V13),
- Rectal swabs (*N* = 2) for PK and multi-omics (V7, V12, and V13),
- Rectal biopsies (*N* = 5) for PK and multi-omics (V7, V12, and V13).

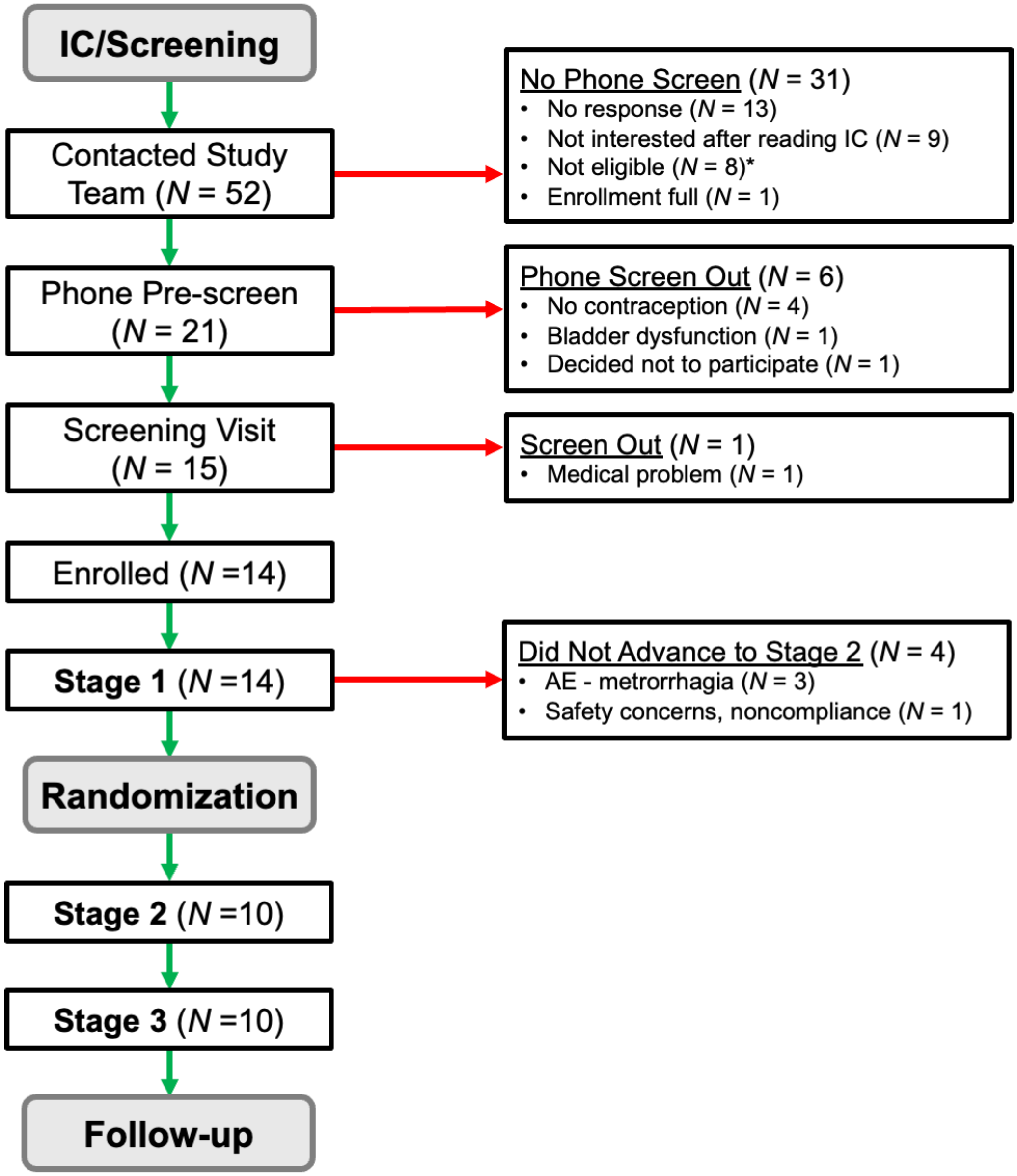

SUPPLEMENTARY FIGURE 1. PrEP-Pod-IVR-02 clinical trial flow diagram, summarizing screening, enrollment, and volunteer participation. *Not eligible based on contact prior to phone pre-screen (2 enrolled in other study, 1 irregular menses, 1 moving away, 1 no contraception, 1 menopause, 1 using NuvaRing, 1 previous hysterectomy).

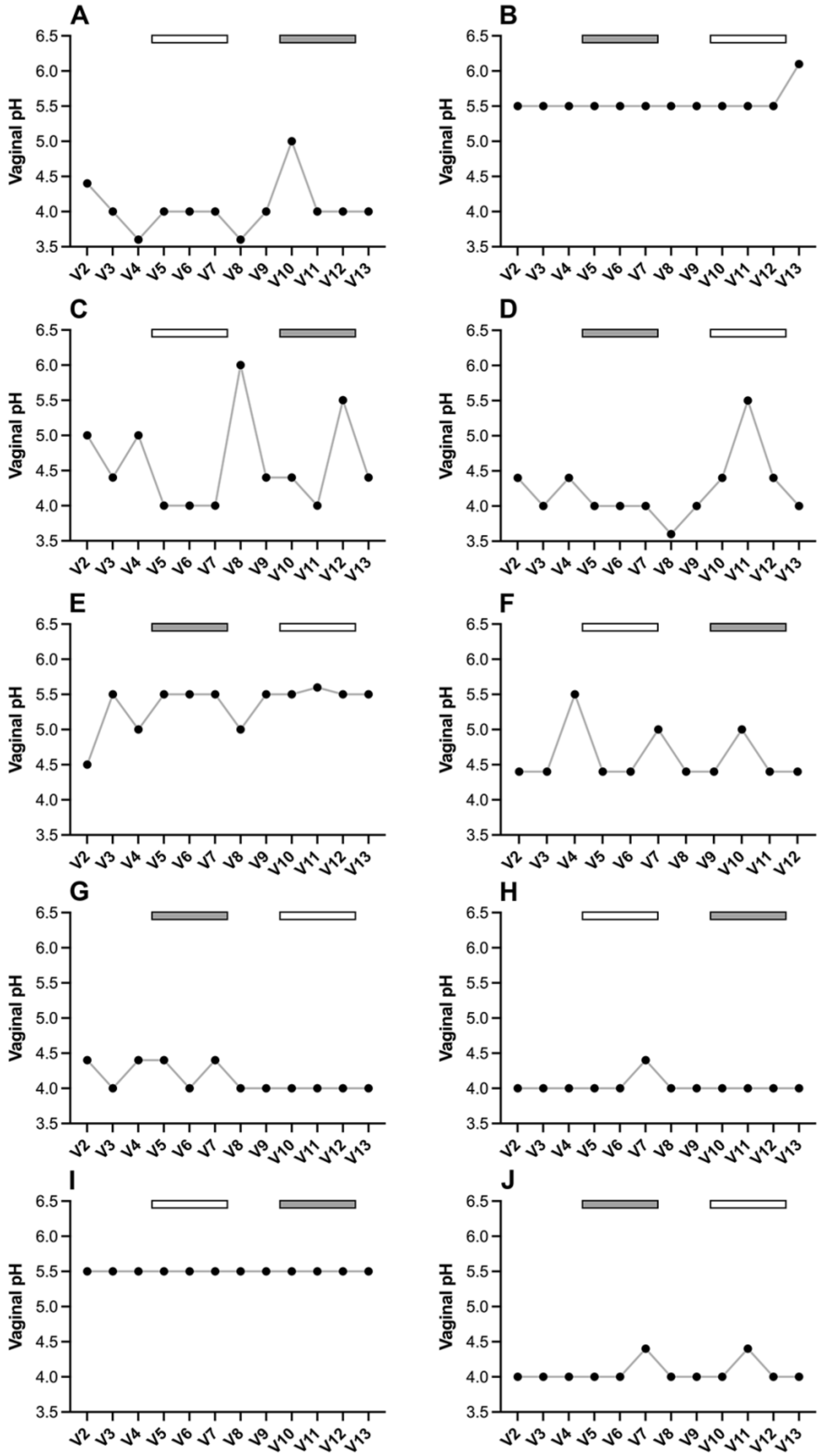

SUPPLEMENTARY FIGURE 2. Vaginal pH of trial participants is not systematically altered in the presence of the TDF-FTC IVR. V1-13 Refer to clinical visits; white horizonal bars identify

periods with placebo (unmedicated) IVR in place; grey horizonal bars identify periods with TDF-FTC IVR in place; (**A**) participant 131-02; (**B**) participant 131-05; (**C**) participant 131-06; (**D**) participant 131-07; (**E**) participant 131-08; (**F**) participant 131-09; (**G**) participant 131-11; (**H**) participant 131-12; (**I**) participant 131-13; (**J**) participant 131-15.

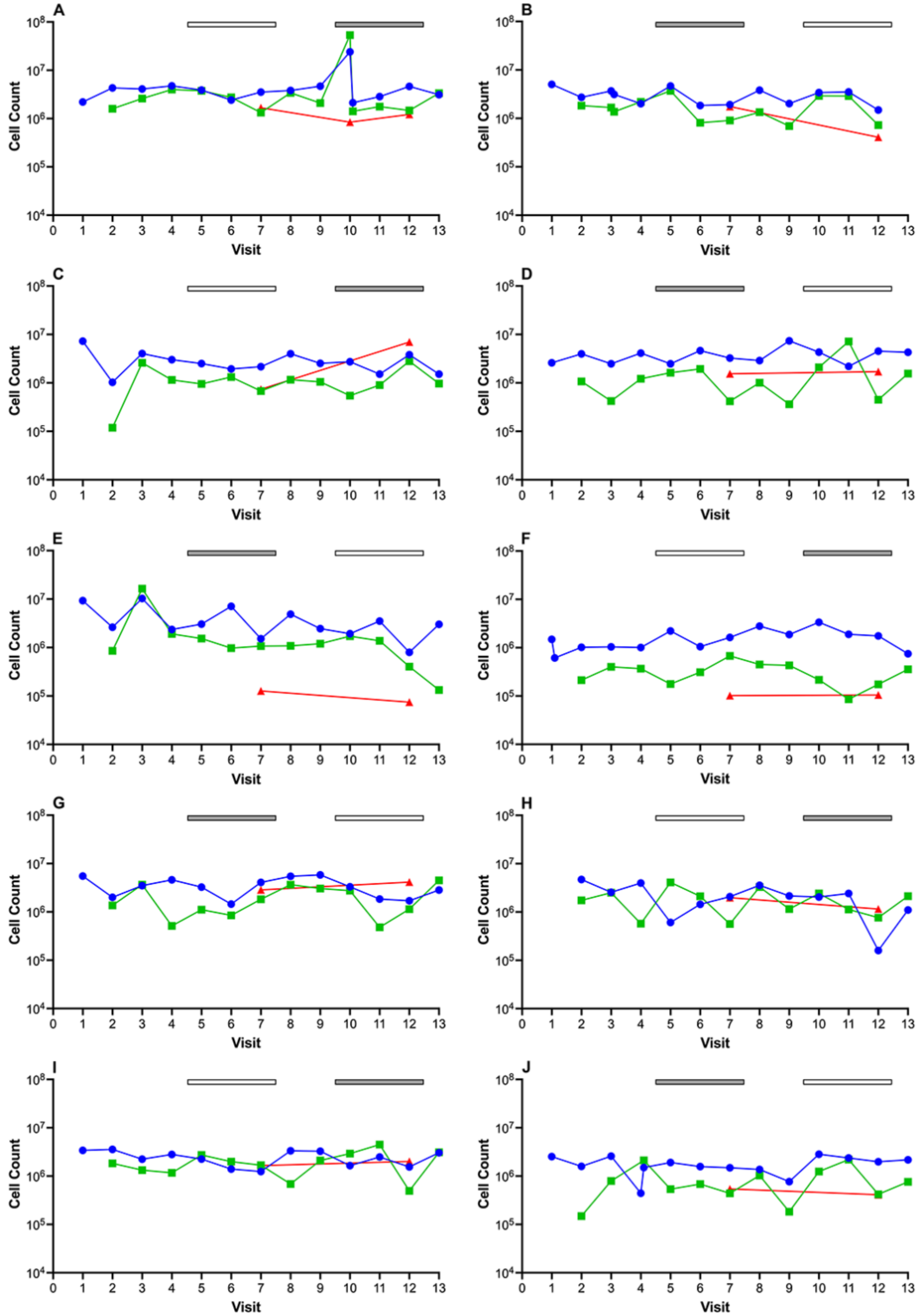

SUPPLEMENTARY FIGURE 3. Cell counts in vaginal samples collected from trial participants; blue circles, vaginal swab; green squares, CVL; red triangles, IVR surface; white horizonal bars identify periods with placebo (unmedicated) IVR in place; grey horizonal bars identify periods with

TDF-FTC IVR in place; (**A**) participant 131-02; (**B**) participant 131-05; (**C**) participant 131-06; (**D**) participant 131-07; (**E**) participant 131-08; (**F**) participant 131-09; (**G**) participant 131-11; (**H**) participant 131-12; (**I**) participant 131-13; (**J**) participant 131-15.

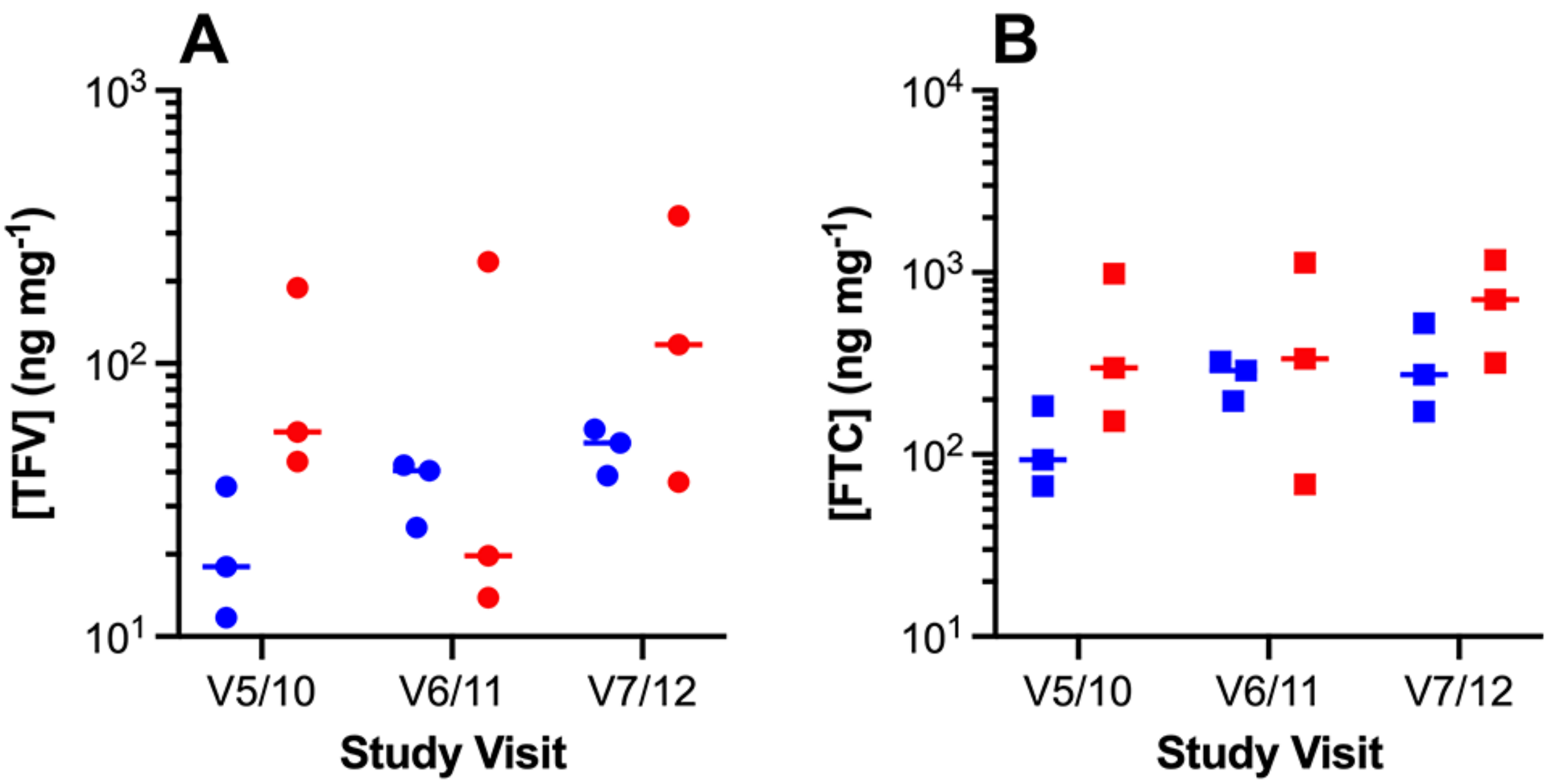

SUPPLEMENTARY FIGURE 4. Cervicovaginal fluid (swab-collected) drug concentrations grouped by VMB. Each symbol represents a single measurement, and medians are represented by horizontal lines; blue data, participant 131-02, -07, -09, *L. crispatus*-dominant (eubiotic); red data, participant 131-05, -08, -13, *G. vaginalis*-dominant (dysbiotic); (**A**) TFV; (**B**) FTC.

SUPPLEMENTARY TABLE 1. All AEs regardless of relationship to study product.

		**Stage 1**	**Stage 2+3**		**Stage 2**		**Stage 3**		**Stage 2+3**	
		Pre-Randomization (Baseline; No IVR) N = 14[1]	Active vs Placebo (crossover) *N* = 10		(1st IVR) *N* = 10; *N* = 5/arm		(2nd IVR) *N* = 10; *N* = 5/arm		per Arm (crossover) *N* = 10; *N* = 5/arm	
			**Placebo**	**Active**	Arm 1 Active	Arm 2 Placebo	Arm 1 Placebo	Arm 2 Active	**Arm 1**	**Arm 2**
**Total AEs**	**84**	**35**	**20**	**29**	**15**	**9**	**11**	**14**	**26**	**23**
Total Gr 1	66	28	14	24	10	7	7	14	17	21
Total Gr 2	17	7	5	5	5	2	3	0	8	2
Total Gr 3	1	0	1[2]	0	0	0	1	0	1	0
Total Gr 4	0	0	0	0	0	0	0	0	0	0

[1]Includes all participants eligible after screening visit
[2]Grade 3 event was a sports injury requiring surgery, unrelated to IVR

SUPPLEMENTARY TABLE 2. MedDRA Categorization of all AEs regardless of relationship to study product.

			Total	Subtotals		
				**Baseline** *N* = 14	**Placebo** *N* = 10	**Active** *N* = 10
**Number of AEs**			**84**	**35**	**20**	**29**
MedDRA SOC	MedDRA Preferred Term	Grade				
Blood and lymphatic system disorders	Lymphadenopathy	Gr 1	**1**		1	
Cardiac disorders	Palpitations	Gr 1	**1**			1
	Tachycardia	Gr 1	**1**	1		
Gastrointestinal disorders	Abdominal pain	Gr 2	**2**	1		1
		Gr 1	**3**		1	2
	Abdominal pain, lower	Gr 1	**1**			1
	Diarrhea	Gr 1	**1**			1
	Nausea	Gr 1	**2**	1	1	
	Vomiting	Gr 1	**2**	2		
Infections and infestations	Bacterial vaginosis	Gr 2	**4**	2	1	1
		Gr 1	**2**	1		1
	Gastroenteritis	Gr 1	**2**		1	1
	Herpes simplex, genital	Gr 1	**1**	1		
	Herpes, oral	Gr 1	**1**			1
	Influenza	Gr 2	**1**	1		
	Upper respiratory tract infection	Gr 2	**1**		1	
		Gr 1	**3**	3		
	Urinary tract infection	Gr 2	**2**		2	
	Vulvovaginal candidiasis	Gr 1	**1**			1
	Vulvovaginitis	Gr 2	**1**			1
		Gr 1	**4**	2		2
Injury, poisoning and procedural complications	Cervix injury	Gr 1	**2**		2	
	Exposure to body fluid	Gr 1	**1**	1		
	Joint injury	Gr 3	**1**		1	
		Gr 1	**1**	1		
	Muscle strain	Gr 1	**1**	1		
	Skin abrasion	Gr 1	**1**			1
	Skin laceration	Gr 2	**1**			1
Investigations	Blood pressure systolic increased	Gr 1	**1**	1		
Nervous system disorders	Headache	Gr 1	**1**		1	

			Total	Subtotals		
				Baseline *N* = 14	Placebo *N* = 10	Active *N* = 10
**Number of AEs**			**84**	**35**	**20**	**29**
MedDRA SOC	MedDRA Preferred Term	Grade				
Product issues	Device expulsion (IUD)	Gr 2	**1**		1	
Renal and urinary disorders	Pollakiuria	Gr 1	**1**			1
	Urethritis noninfective	Gr 1	**3**	3		
Reproductive system and breast disorders	Adnexa uteri mass	Gr 1	**1**			1
	Breast tenderness	Gr 1	**1**	1		
	Genital ulceration	Gr 1	**1**	1		
	Menorrhagia	Gr 2	**1**	1		
		Gr 1	**1**			1
	Menometrorrhagia	Gr 2	**1**	1		
		Gr 1	**1**	1		
	Metrorrhagia	Gr 2	**1**	1		
		Gr 1	**5**	2	1	2
	Ovarian cyst	Gr 1	**3**	1		2
	Uterine spasm	Gr 1	**1**			1
	Vaginal discharge	Gr 1	**7**	2	3	2
	Vaginal hemorrhage	Gr 1	**1**		1	
	Vulvovaginal burning sensation	Gr 1	**1**	1		
	Vulvovaginal pain	Gr 1	**1**			1
Respiratory, thoracic and mediastinal disorders	Nasal congestion	Gr 1	**1**		1	
	Rhinitis allergic	Gr 2	**1**			1
		Gr 1	**1**			1
Skin and subcutaneous tissue disorders	Dermatitis allergic	Gr 1	**1**	1		
	Intertrigo	Gr 1	**1**		1	

SUPPLEMENTARY TABLE 3. MedDRA Categorization of all AEs related to IVR use.

MedDRA SOC	MedDRA Preferred Term	Grade	Placebo (8)	Active (11)	Total (19)
Blood and lymphatic system disorders	Lymphadenopathy	Gr 1	1		**1**
Gastrointestinal disorders	Abdominal pain	Gr 1	1	2	**3**
	Abdominal pain, lower	Gr 1		1	**1**
Infections and infestations	Bacterial vaginosis	Gr 2	1	1	**2**
	Vulvovaginal candidiasis	Gr 1		1	**1**
	Vulvovaginitis	Gr 2		1	**1**
		Gr 1		1	**1**
Product issues	Device expulsion[1]	Gr 2	1		**1**
Renal and urinary disorders	Pollakiuria	Gr 1		1	**1**
Reproductive system and breast disorders	Menorrhagia	Gr 1		1	**1**
	Metrorrhagia	Gr 1	1		**1**
	Vaginal discharge	Gr 1	3	2	**5**

[1]The device expelled was the participant's IUD, not the study product
